# Real‐World Associations of Renin–Angiotensin–Aldosterone System Inhibitor Dose, Hyperkalemia, and Adverse Clinical Outcomes in a Cohort of Patients With New‐Onset Chronic Kidney Disease or Heart Failure in the United Kingdom

**DOI:** 10.1161/JAHA.119.012655

**Published:** 2019-11-12

**Authors:** Cecilia Linde, Ameet Bakhai, Hans Furuland, Marc Evans, Phil McEwan, Daniel Ayoubkhani, Lei Qin

**Affiliations:** ^1^ Heart and Vascular Theme Karolinska University Hospital and Karolinska Institutet Stockholm Sweden; ^2^ Department of Cardiology Royal Free Hospital London United Kingdom; ^3^ Department of Nephrology Uppsala University Hospital Uppsala Sweden; ^4^ Diabetes Resource Centre Llandough Hospital Cardiff United Kingdom; ^5^ Health Economics and Outcomes Research Ltd. Cardiff United Kingdom; ^6^ Global Health Economics AstraZeneca Gaithersburg MD

**Keywords:** chronic kidney disease, heart failure, hyperkalemia, major adverse cardiac event, renin–angiotensin system, Heart Failure, ACE/Angiotension Receptors/Renin Angiotensin System, Nephrology and Kidney, Treatment, Mortality/Survival

## Abstract

**Background:**

Dosing of renin–angiotensin–aldosterone system inhibitors (RAASi) may be modified to manage associated hyperkalemia risk; however, this approach could adversely affect cardiorenal outcomes. This study investigated real‐world associations of RAASi dose, hyperkalemia, and adverse clinical outcomes in a large cohort of UK cardiorenal patients.

**Methods and Results:**

This observational study included RAASi‐prescribed patients with new‐onset chronic kidney disease (n=100 572) or heart failure (n=13 113) first recorded between January 2006 and December 2015 in Clinical Practice Research Datalink and linked Hospital Episode Statistics databases. Odds ratios associating hyperkalemia and RAASi dose modification were estimated using logistic generalized estimating equations with normal (<5.0 mmol/L) serum potassium level as the reference category. Patients with serum potassium ≥5.0 mmol/L had higher risk of RAASi down‐titration (adjusted odds ratios, chronic kidney disease: 1.79 [95% CI, 1.64–1.96]; heart failure: 1.33 [95% CI, 1.08–1.62]). Poisson models were used to estimate adjusted incident rate ratios of adverse outcomes based on total RAASi exposure (<50% and ≥50% of the guideline‐recommended RAASi dose). Incidence of major adverse cardiac events and mortality was consistently higher in the lower dose group (adjusted incident rate ratios: chronic kidney disease: 5.60 [95% CI, 5.29–5.93] for mortality and 1.60 [95% CI, 1.55–1.66] for nonfatal major adverse cardiac events; heart failure: 7.34 [95% CI, 6.35–8.48] for mortality and 1.85 [95% CI, 1.71–1.99] for major adverse cardiac events).

**Conclusions:**

The results of this real‐world analysis highlight the potential negative impact of suboptimal RAASi dosing and the need for strategies that allow patients to be maintained on appropriate therapy, avoiding RAASi dose modification or discontinuation.

**Clinical Perspective**

**What Is New?**

- This study aimed to estimate real‐world associations of renin–angiotensin–aldosterone system inhibitor (RAASi) dose, hyperkalemia, and adverse clinical outcomes in a large cohort of UK patients with new‐onset chronic kidney disease or heart failure.
- Underprescribing of RAASi relative to guideline‐recommended dosing was ubiquitous among the study population and increased following hyperkalemia events.
- The incidence of major adverse cardiac events and mortality was consistently higher in patients who were underprescribed RAASi (ie, receiving <50% of the recommended dose over the majority of their follow‐up).

**What Are the Clinical Implications?**

- Results of this study emphasize the potential negative impact of suboptimal RAASi dosing and, consequently, the need for strategies that allow patients to be maintained on appropriate therapy, avoiding RAASi dose modification or discontinuation.
- We propose that European Society of Cardiology–recommended RAASi doses for patients with heart failure may be generalizable to chronic kidney disease patients in the absence of chronic kidney disease–specific recommendations.

## Introduction

The renin–angiotensin–aldosterone system (RAAS) plays a key role in the regulation of blood volume, blood pressure, and cardiovascular function.^1^ Modification of the RAAS through the use of RAAS inhibitors (RAASi), such as angiotensin‐converting enzyme inhibitors (ACEIs), angiotensin receptor blockers (ARBs), and mineralocorticoid receptor antagonists (MRAs), is an important therapeutic option for the treatment of numerous cardiorenal conditions, reducing progression of chronic kidney disease (CKD), improving heart function and reducing cardiovascular morbidity and mortality.^2^, ^3^, ^4^, ^5^, ^6^, ^7^, ^8^ However, RAASi use is known to reduce potassium (K^+^) excretion and increase the risk of hyperkalemia in an already vulnerable population.^9^, ^10^

Hyperkalemia is a potentially life‐threatening electrolyte imbalance, defined as a serum/plasma K^+^ level above the normal physiological range of 3.5 to 5.0 mmol/L,^11^ that can induce electrophysiological disturbances, potentially leading to cardiac arrhythmias, cardiac arrest, and sudden death.^12^, ^13^, ^14^ The long‐term management of serum K^+^ often requires down‐titration or discontinuation of RAASi, an approach supported by UK clinical practice guidelines: the National Institute for Health and Care Excellence (NICE) recommends not offering RAASi to CKD patients whose pretreatment serum K^+^ is >5.0 mmol/L and discontinuing them in patients whose serum K^+^ reaches ≥6.0 mmol/L,^6^ whereas guidelines from the UK Renal Association advocate cautious use or avoidance of drugs that impair K^+^ elimination (including RAASi) and recommend discontinuation following a hyperkalemia episode.^15^ In routine clinical practice, underdosing of RAASi has been found to be common among both heart failure (HF)^16^, ^17^, ^18^, ^19^ and CKD^19^ patients, with RAASi down‐titration or discontinuation following 22% to 47% of hyperkalemia events in cardiorenal patients.^19^, ^20^ RAASi down‐titration or discontinuation in these patients has been shown to be associated with adverse clinical outcomes, including death.^16^, ^18^, ^19^

The link between RAASi use and hyperkalemia, and evidence of poor outcomes associated with suboptimal RAASi dosing have both been reported previously.^16^, ^17^, ^18^, ^20^ However, the relationships of RAASi dose, hyperkalemia, and clinical outcomes in cardiorenal patients have not been fully described. Nevertheless, outcomes in RAASi‐treated patients are of high potential interest to cardiologists, nephrologists, general practitioners, and the general medical community, given the widespread applications of RAASi in cardiorenal medicine. Our study aimed to address this evidence gap by estimating real‐world associations of RAASi dose, hyperkalemia, and adverse clinical outcomes in a large cohort of UK patients with new‐onset CKD or HF. More specifically, the study addressed 2 objectives: to investigate the associations between the incidence of hyperkalemia and changes to RAASi dose (down‐titration or discontinuation) and to assess real‐world RAASi dosing and its association with adverse clinical outcomes.

## Methods

Because of the sensitive nature of the data collected for this study, requests to access the data set by qualified researchers trained in human subject confidentiality protocols may be sent to the Clinical Practice Research Datalink (CPRD) at enquiries@cprd.com.

### Study Population

Adults (aged ≥18 years) with new‐onset HF or nondialysis CKD stage ≥3 who were prescribed RAASi treatment between January 1, 2006, and December 31, 2015, were included in the study. RAASi considered included specific ACEIs, ARBs, and MRAs for which dosage was recommended by the European Society of Cardiology (ESC) 2016 guidelines for the treatment of HF^3^; recommended dosage was applied to both HF and CKD patients, given the lack of specific guidelines on RAASi dosing in CKD. For HF patients, no information of left ventricular ejection fraction was available.

Patient data were obtained from the CPRD—a source of longitudinal, coded, anonymized electronic health records from a UK‐wide network of >1100 primary care practices^21^ considered to be broadly representative of patients within the United Kingdom. The database includes information on >10 million currently registered patients, with linked secondary care data from Hospital Episode Statistics (HES) available for a subset of patients from participating practices in England.^21^, ^22^

Each patient's follow‐up period was defined as time from the date of their first RAASi prescription following the initial CKD or HF record (or first CKD or HF record date for those not on RAASi) during the study period until death, loss to follow‐up, or end of the study, whichever occurred first. CKD was defined as (1) a READ code for CKD stage ≥3, (2) an *International Classification of Diseases, Tenth Revision* (*ICD‐10*) code for CKD stage ≥3 obtained from HES data linked to the CPRD, or (3) an estimated glomerular filtration rate <60 mL/min per 1.73 m^2^. HF was defined as (1) a READ code for HF or (2) an *ICD‐10* code for HF obtained from HES data linked to the CPRD. The nature of the first event recorded during the study period (CKD or HF) determined patient classification to the respective cohorts. Patients were excluded from the study if they had a history of CKD or HF recorded within the 5 years before the study period (ie, between January 1, 2001, and December 31, 2005) or if information on treatment dose received was inadequate. In addition, CKD patients were excluded if their first CKD event during the study period was dialysis or a kidney transplant. Patient characteristics were described at baseline, that is, at the time of each patient's first RAASi prescription after their CKD or HF event. Rather than relying solely on measurements taken on the baseline date, a look‐forward period of 12 months was used for baseline patient characteristics; the measurement taken closest to the baseline date within a 12‐month window after that date was used as the baseline value.

The study was approved by the UK Independent Scientific Advisory Committee for Medicines and Healthcare Products Regulatory Agency database research on December 15, 2016 (study protocol 16_223R). Informed consent from individual patients was not required.

### Statistical Analysis

All statistical analyses were performed using R v3.4.2,^14^ with the exception of the multinomial logistic regression model for discontinuation and down‐titration of RAASi, which was performed in SAS v9.4.

RAASi down‐titration was defined as a reduction in RAASi dose between consecutive prescriptions with a gap of <90 days between the end of one prescription (expected end based on prescribing date, dosing strategy, and number of tablets) and start of the next. Treatment discontinuation was defined by the cessation of RAASi prescriptions or a >90‐day interval between consecutive prescriptions for the same therapy. A multinomial multivariable logistic regression model was used to estimate adjusted odds ratios (ORs) of RAASi down‐titration and discontinuation, comparing patients with and without hyperkalemia (serum K^+^ ≥5.0 versus <5.0 mmol/L). Serum K^+^ thresholds of ≥5.5 mmol/L and ≥6.0 mmol/L were investigated in sensitivity analyses.

When estimating associations between RAASi dose and death (all‐cause mortality) and nonfatal MACE (a composite of arrhythmia, HF, myocardial infarction, and stroke^23^), patient follow‐up was separated into quarterly time windows, and only those windows spent on RAASi treatment were included in the analysis. Mean RAASi dose within each quarter was calculated as the mean dose across all therapies within each quarter, weighted by the proportion of time within each quarter that a patient was on each therapy (calculated as specified in Data S1 and Figure S1). Crude event rates were calculated per 1000 patient‐years of exposure time stratified by dose. Cumulative incidence curves were fitted using Cox models adjusted using baseline characteristics, stratified by patients who spent the majority of their follow‐up (defined at ≥75% of quarters) on <50% or ≥50% of ESC guideline‐recommended RAASi dose.^3^

Adjusted incident rate ratios (IRRs) were used to further explore associations between adverse outcomes and RAASi dose within time‐updated intervals, comparing patients on <50% and ≥50% of ESC guideline‐recommended RAASi dose. IRRs were derived from Poisson regression models estimated by generalized estimating equations to align with a prior publication by Furuland et al,^24^ which described the development of risk equations to predict the risk of adverse clinical outcomes using time‐varying serum K^+^ levels in CKD patients from the CPRD. Additional covariates were included to control for patient characteristics and clinical histories (eg age, sex, smoking status, clinical history, medication use, and laboratory values). Variable reduction, which minimizes the number of covariates included in a statistical model while maintaining power, was initially performed by the LASSO (least absolute shrinkage and selection operator) method, with final variable selection being informed by clinical interpretability and the quasi information criterion.

Multiple imputation^25^, ^26^ and last observation carried forward were used to accommodate missing clinical measurements. Five imputed data sets were produced, and model coefficients and their standard errors (SEs) were pooled across data sets based on Rubin Rules,^27^ to capture the variance of the coefficients both within and between the imputed data sets. Multiple imputation was performed on all clinical variables with all candidate covariates and outcome variables from the analysis models, using the chained equations method, as implemented in the R package “mice.”^28^ A schematic of the multiple imputation process is presented in Figures S2 and S3. The performance of missing data entry using multiple imputation was verified by performing a sensitivity analysis using a 3‐month (data not shown) as opposed to 12‐month (Table S1) look‐forward period for baseline patient characteristics. Although the percentage of missing baseline data was considerably higher with the shorter look‐forward period, the model results changed very little (data not shown), suggesting that imputation methods were appropriate and the proportion of missing data did not substantially affect the results of the analysis.

Further details surrounding the modeling methodology are outlined in Data S1, and detailed outputs of the model are listed in Tables S2 through S7.

P.M. and D.A. had full access to all the data in the study and take responsibility for its integrity and the data analysis.

## Results

### Baseline Patient Disposition

**Figure 1:**
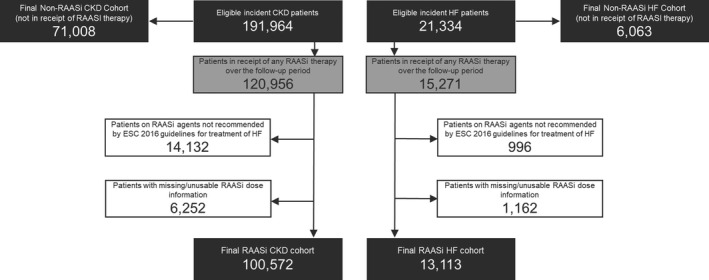
Study participation flow diagram. CKD indicates chronic kidney disease; ESC, European Society of Cardiology; HF, heart failure; RAASi, renin–angiotensin–aldosterone system inhibitors.

Data for 191 964 CKD patients and 21 334 HF patients were identified: of these, 14 132 CKD and 996 HF patients were excluded because no ESC‐recommended^3^ dose was available for their received treatments; a further 6252 CKD and 1162 HF patients were excluded because of missing or unusable RAASi dose information. Patients who did not receive any RAASi therapy over the follow‐up period (71 008 CKD and 6063 HF patients, respectively, hereby referred to as *non‐RAASi*) were included in some analyses for comparative purposes. This resulted in final RAASi cohort sizes of 100 572 CKD and 13 113 HF patients (Figure 1). More details on patient attrition (Table S8) and missing data are provided in Data S1, and baseline patient characteristics of the HF and CKD cohorts stratified by ESC‐recommended dose are listed in Table 1. In addition, baseline characteristics were compared between the RAASi cohort and the patients who were excluded from the study because of receiving a medication for which a recommended dose was not specified in the ESC guidelines or because of missing or unusable RAASi dose information; this comparison is provided in Table S9.

**Table 1:** Baseline Patient Demographics, Clinical Characteristics, and Clinical Histories of the CKD and HF Cohorts Stratified by the RAASi Dose Achieved During the Majority of Patients’ Follow‐Up Period

Variable	CKD Cohort				HF Cohort			
	RAASi Dose Achieved During the Majority (≥75%) of Follow‐Up				RAASi Dose Achieved During the Majority (≥75%) of Follow‐Up			
	Non‐RAASi (n=71 008)	<50% of ESC‐Recommended Dose (n=27 935)^a^	≥50% of ESC‐Recommended Dose (n=26 596)^a^	*P* Value (ANOVA/χ^2^)	Non‐RAASi (n=6063)	<50% of ESC‐Recommended Dose (n=4568)^a^	≥50% of ESC‐Recommended Dose (n=2758)^a^	*P* Value (ANOVA/χ^2^)
Baseline^b^ patient demographics and clinical characteristics								
Age, y, mean (SD)	71.77 (14.20)	74.84 (10.79)	71.43 (10.36)	<0.01^c^	74.97 (15.83)	75.5 (12.48)	68.93 (12.74)	<0.01^c^
Female	46 320 (65.22)	16 492 (59.04)	14 776 (55.56)	<0.01^c^	3007 (49.59)	2015 (44.11)	906 (32.85)	<0.01^c^
Current smoker	10 599 (14.92)	3681 (13.18)	3705 (13.93)	<0.01^c^	702 (11.58)	807 (17.67)	582 (21.10)	<0.01^c^
BMI, kg/m^2^, mean (SD)	27.02 (5.59)	28.28 (5.69)	29.80 (5.88)	<0.01^c^	26.49 (6.90)	27.49 (6.42)	29.73 (6.66)	<0.01^c^
SBP, mm Hg, mean (SD)	134.33 (18.36)	138.58 (19.92)	143.45 (19.90)	<0.01^c^	128.17 (20.75)	125.91 (20.70)	132.98 (21.92)	<0.01^c^
eGFR, mL/min/1.73 m^2^, mean (SD)	51.43 (8.47)	51.23 (10.45)	51.98 (8.65)	<0.01^c^	63.38 (19.52)	64.7 (17.74)	67.75 (15.40)	<0.01^c^
Serum potassium, mEq/L, mean (SD)	4.44 (0.53)	4.49 (0.52)	4.49 (0.51)	<0.01^c^	4.28 (0.60)	4.42 (0.55)	4.42 (0.51)	<0.01^c^
Serum phosphorus, mEq/L, mean (SD)	1.14 (1.00)	1.15 (1.59)	1.11 (0.20)	0.18	1.17 (0.26)	1.16 (0.23)	1.15 (0.21)	0.46
Clinical history within 5 y before initial CKD/HF diagnosis								
Diabetes mellitus	5234 (7.37)	4410 (15.79)	5843 (21.97)	<0.01^c^	715 (11.79)	637 (13.94)	524 (19.00)	<0.01^c^
MI	872 (1.23)	1467 (5.25)	1090 (4.10)	<0.01^c^	234 (3.86)	532 (11.65)	388 (14.07)	<0.01^c^
PVD	1059 (1.49)	749 (2.68)	771 (2.90)	<0.01^c^	121 (2.00)	159 (3.48)	83 (3.01)	<0.01^c^
Stroke	3835 (5.40)	1817 (6.50)	1426 (5.36)	<0.01^c^	352 (5.80)	329 (7.20)	135 (4.89)	0.01^c^
Arrhythmia	4315 (6.08)	2663 (9.53)	2020 (7.60)	<0.01^c^	830 (13.69)	1144 (25.04)	651 (23.60)	<0.01^c^
CPD	6199 (8.73)	2818 (10.09)	2526 (9.50)	<0.01^c^	562 (9.27)	721 (15.78)	398 (14.43)	<0.01^c^
Metastatic tumor	1640 (2.31)	599 (2.14)	508 (1.91)	<0.01^c^	89 (1.47)	93 (2.04)	50 (1.81)	0.08
Rheumatic disease	2307 (3.25)	942 (3.37)	732 (2.75)	<0.01^c^	126 (2.08)	135 (2.96)	68 (2.47)	0.02^c^
Peptic ulcer	568 (0.80)	239 (0.86)	211 (0.79)	0.64	55 (0.91)	54 (1.18)	23 (0.83)	0.24
Cancer	7074 (9.96)	2513 (9.00)	2068 (7.78)	<0.01^c^	516 (8.51)	527 (11.54)	220 (7.98)	<0.01^c^
Baseline^b^ medication usage								
β‐Blockers	11 332 (15.96)	8737 (31.28)	8864 (33.33)	<0.01^c^	999 (16.47)	2636 (57.71)	1877 (68.06)	<0.01^c^
Statins	19 163 (26.98)	15 562 (55.71)	15 734 (59.16)	<0.01^c^	1040 (17.15)	2354 (51.53)	1731 (62.76)	<0.01^c^
Bronchodilators	7329 (10.32)	3377 (12.09)	2608 (9.81)	<0.01^c^	727 (11.99)	960 (21.02)	474 (17.19)	<0.01^c^
Diuretics	16 736 (23.57)	12 911 (46.22)	13 998 (52.63)	<0.01^c^	2001 (33.00)	3691 (80.80)	2117 (76.76)	<0.01^c^
NSAIDs	9589 (13.50)	2441 (8.74)	2535 (9.53)	<0.01^c^	283 (4.67)	217 (4.75)	131 (4.75)	0.97
Calcium channel blockers	11 778 (16.58)	7775 (27.83)	10 547 (39.66)	<0.01^c^	627 (10.34)	522 (11.43)	589 (21.36)	<0.01^c^
OADs	2869 (4.04)	3226 (11.55)	4186 (15.74)	<0.01^c^	226 (3.73)	450 (9.85)	392 (14.21)	<0.01^c^
Insulin	765 (1.08)	795 (2.85)	1088 (4.09)	<0.01^c^	61 (1.01)	109 (2.39)	93 (3.37)	<0.01^c^

Data are shown as n (%) except as noted. ANOVA and χ^2^ test were used to evaluate differences between HbA1c groups for continuous and categorical variables, respectively. BMI indicates body mass index; CKD, chronic kidney disease; CPD, cardiopulmonary disease; eGFR, estimated glomerular filtration rate; ESC, European Society of Cardiology; HF, heart failure; MI, myocardial infarction; OADs, oral antidiabetics; PVD, peripheral vascular disease; RAASi, renin–angiotensin–aldosterone system inhibitors; SBP, systolic blood pressure.

a The numbers of patients at <50% and ≥50% of the recommended dose do not add up to the total cohort size because patients who spent most of their time on 0% dose and those who do not have a clear majority of time spent at a given dose level are not shown in the table but are included in the total cohort.

b Baseline for RAASi patients is time of each patient's first RAASi prescription after their CKD or HF event and for non‐RAASi patients is time of first CKD or HF event.

c *P*<0.05.

### RAASi Doses Prescribed During Follow‐Up

Frequency of individual RAASi use and the number of prescriptions >50% of the ESC guideline‐recommended dose for both cohorts are shown in Table 2. ACEIs were the most commonly prescribed RAASi type among both CKD (76.4%) and HF (73.5%) patients. ARBs were more frequently prescribed in CKD than HF patients (32.1% versus 25.1%), whereas the reverse was observed for MRAs (9.6% versus 43.0%). Across all RAASi, ramipril was most frequently prescribed (48.7% of CKD patients and 60.0% of HF patients). Nearly all MRA prescriptions (91.2% and 90.5% for the CKD and HF cohorts, respectively) included doses >50% of the guideline‐recommended dose, compared with just over a third of ARB prescriptions (39.8% and 35.1% for the CKD and HF cohorts, respectively). The mean duration of a discontinuation was 867.02 days (SD: 790.01) for the CKD cohort and 690.33 days (SD: 655.55) for the HF cohort.

**Table 2:** Guideline‐Recommended Doses of RAASi During Follow‐Up

Drug	Guideline‐Recommended Daily Dose (mg)	CKD Cohort, n (%)			HF Cohort, n (%)		
		Prescriptions	Prescriptions ≥50% Guideline‐Recommended Dose	Patients	Prescriptions	Prescriptions ≥50% Guideline‐Recommended Dose	Patients
ACEIs	···	2 558 015 (66.81)	1 628 928 (63.68)	76 788 (76.35)	245 524 (54.39)	150 468 (61.28)	9636 (73.48%)
Ramipril	10	1 518 468 (39.66)	1 001 162 (65.93)	48 960 (48.68)	195 053 (43.21)	120 869 (61.97)	7874 (60.05%)
Lisinopril	20	827 171 (21.60)	515 919 (62.37)	24 820 (24.68)	40 869 (9.05)	25 339 (62.00)	1643 (12.53%)
Enalapril maleate	40	198 480 (5.18)	104 383 (52.59)	5461 (5.43)	9160 (2.03)	4048 (44.19)	362 (2.76%)
Captopril	150	13 896 (0.36)	7464 (53.71)	469 (0.47)	442 (0.10)	212 (47.96)	26 (0.20%)
ARBs	···	1 089 808 (28.46)	433 446 (39.77)	32 297 (32.11)	90 935 (20.15)	31 914 (35.10)	3291 (25.10%)
Candesartan cilexetil	32	517 665 (13.52)	181 537 (35.07)	16 526 (16.43)	55 597 (12.32)	19 285 (34.69)	1990 (15.18%)
Losartan K^+^	150	473 597 (12.37)	205 895 (43.47)	16 448 (16.35)	27 633 (6.12)	9788 (35.42)	1315 (10.03%)
Valsartan	320	98 546 (2.57)	46 014 (46.69)	3120 (3.10)	7705 (1.71)	2841 (36.87)	271 (2.07%)
MRAs	···	181 199 (4.73)	165 280 (91.21)	9687 (9.63)	114 933 (25.46)	104 021 (90.51)	5640 (43.01%)
Spironolactone	50	168 822 (4.41)	153 765 (91.08)	9252 (9.20)	99 159 (21.97)	88 965 (89.72)	5126 (39.09%)
Eplerenone	50	12 377 (0.32)	11 515 (93.04)	666 (0.66)	15 774 (3.49)	15 056 (95.45)	779 (5.94%)
Total	···	3 829 022	2 227 654 (58.18)	100 572	451 392	286 403 (63.45)	13 113

ACEI indicates angiotensin‐converting enzyme inhibitor; ARB, angiotensin II receptor blocker; CKD, chronic kidney disease; HF, heart failure; MRA, mineralocorticoid receptor antagonist; RAASi, renin–angiotensin–aldosterone system inhibitors.

### Associations Between Hyperkalemia and RAASi Titration

**Figure 2:**
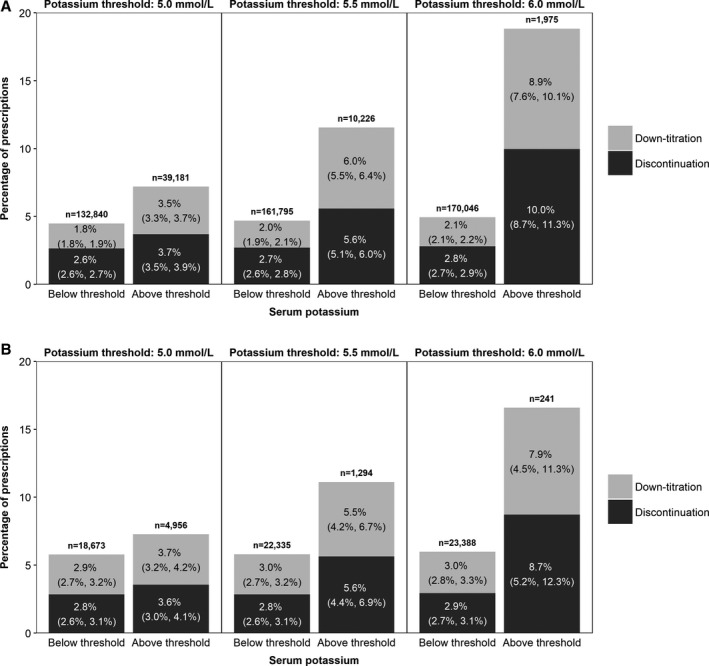
Dose modification of renin–angiotensin–aldosterone system inhibitor prescriptions ending within 7 days of a serum potassium (K^+^) measurement, stratified by serum K^+^ threshold for CKD patients (**A**) and HF patients (**B**). Percentages of prescriptions that were maintained or up‐titrated are not shown; prescription numbers include all prescriptions, regardless of dose. The percentage of down‐titrated or discontinued prescriptions is shown on the bars, alongside the 95% CI (in brackets).

Due to the potential causal association between RAASi use and acute kidney injury,^29^ prescriptions issued after any renal failure event were excluded from the discontinuation and down‐titration analysis. RAASi down‐titrations and discontinuations were more common for patients with hyperkalemia compared with those without, and there appeared to be a linear correlation between increasing hyperkalemia severity (ie, higher K^+^ threshold used to define hyperkalemia) and the odds of down‐titration or discontinuation (Figure 2). At a K^+^ threshold of 5.0 mmol/L, 3.5% (95% CI, 3.3–3.7%) of prescriptions in the CKD cohort were down‐titrated and 3.7% (95% CI, 3.5–3.9%) were discontinued for patients with serum K^+^ above threshold compared with 1.8% (95% CI, 1.8–1.9%) and 2.6% (95% CI, 2.6–2.7%), respectively, for those below threshold. Similar results were observed in the HF cohort, in which 3.7% (95% CI, 3.2–4.2%) of prescriptions were down‐titrated and 3.6% (95% CI, 3.0–4.1%) were discontinued for patients with serum K^+^ ≥5.0 mmol/L compared with 2.9% (95% CI, 2.7–3.2%) and 2.8% (95% CI, 2.6–3.1%), respectively, for those with serum K^+^ <5.0 mmol/L. The percentage of discontinued and down‐titrated prescriptions increased with increasing K^+^ threshold used to define hyperkalemia (Figure 2).

**Figure 3:**
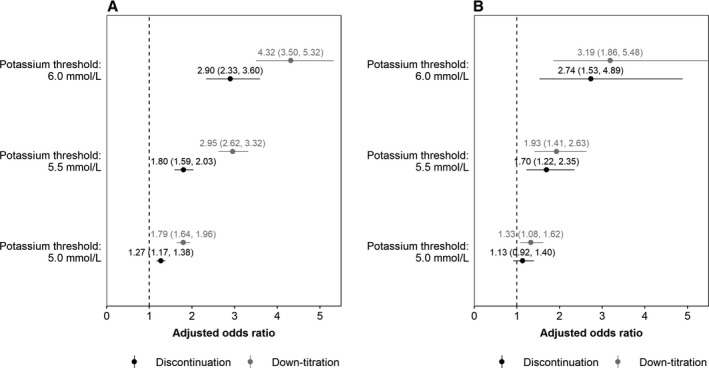
Adjusted odds ratios (and 95% CIs) for dose modification of renin–angiotensin–aldosterone system inhibitors, stratified by serum potassium (K^+^) threshold for CKD patients (**A**) and HF patients (**B**). An odds ratio of 1 (dotted line) indicates the odds of discontinuation or down‐titration under the defined threshold.

After adjusting for covariates, a positive, statistically significant association was found between hyperkalemia and RAASi down‐titration (Figure 3). Adjusted ORs for down‐titration comparing patients with serum K^+^ ≥5.0 mmol/L and those with K^+^ levels below this threshold (reference category) were 1.79 (95% CI, 1.64–1.96) in the CKD cohort and 1.33 (95% CI, 1.08–1.62) in the HF cohort, increasing to 4.32 (95% CI, 3.50–5.32) and 3.19 (95% CI, 1.86–5.47) in the CKD and HF cohorts, respectively, at the more severe hyperkalemia threshold of 6.0 mmol/L.

The pattern of estimated associations between RAASi discontinuation and hyperkalemia was similar, although the associations were weaker than for down‐titration, and in the HF cohort, CIs around the ORs at different K^+^ levels overlapped (Figure 3). Comparing patients with serum K^+^ ≥5.0 mmol/L with those below the threshold, adjusted ORs for discontinuation were 1.27 (95% CI, 1.17–1.38) in the CKD cohort and 1.13 (95% CI, 0.92–1.40) in the HF cohort. When a K^+^ threshold of ≥6.0 mmol/L was considered, adjusted ORs for discontinuation increased to 2.90 (95% CI, 2.33–3.60) and 2.74 (95% CI, 1.53–4.89) in the CKD and HF cohorts, respectively.

### Associations Between RAASi Dosing and Adverse Clinical Outcomes

**Figure 4:**
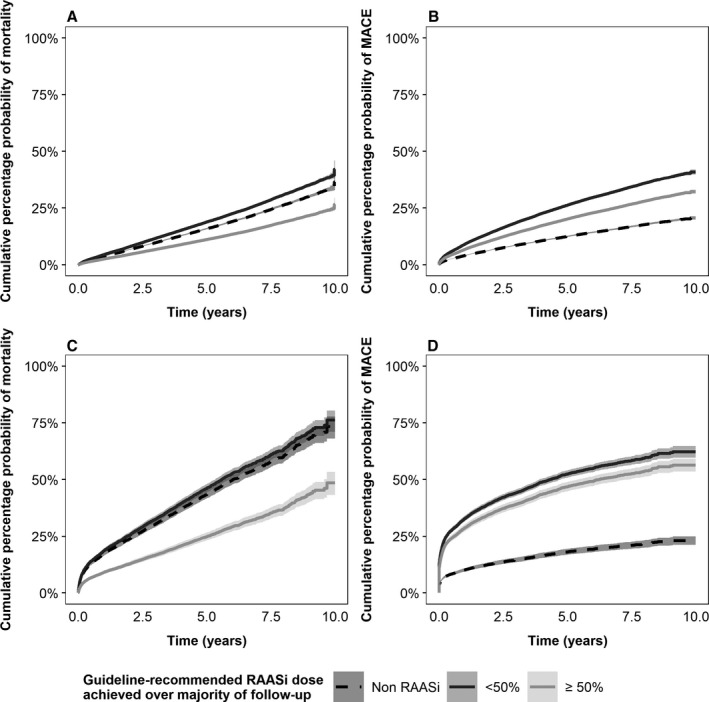
Cox models (and 95% CIs) of cumulative incidence of mortality for CKD patients (**A**), major adverse cardiac events (MACE) for CKD disease patients (**B**), mortality for HF patients (**C**), and MACE for HF patients (**D**). Each graph compares 3 patient groups: (1) those who did not receive any RAASi therapy during the follow‐up and patients who spent the majority of their follow‐up on either (2) <50% or (3) ≥50% of the European Society of Cardiology guideline‐recommended dose. RAASi indicates renin–angiotensin–aldosterone system inhibitors.

The patterns of Cox‐adjusted cumulative incidence of mortality from baseline were similar between CKD and HF cohorts: mortality was consistently lower in patients who spent most of their follow‐up on ≥50% of the ESC guideline‐recommended dose compared with those mostly receiving <50% of the recommended dose, whereas patients who did not receive any RAASi therapy over the follow‐up period had a slightly lower incidence of mortality than patients on <50% dose (Figure 4). In both CKD and HF cohorts, cumulative incidence of nonfatal major adverse cardiac events (MACE) was lowest in non‐RAASi patients and highest in patients taking <50% of the guideline‐recommended dose.

Event rates for all‐cause mortality and MACE in the CKD and HF cohorts stratified by the dose received within the interval where the event occurred are summarized in Table 3. In the CKD cohort, mortality occurred at a rate of 57.7 (95% CI, 56.6–58.9) deaths per 1000 patient‐years for patients on <50% of guideline‐recommended dose compared with 7.2 (95% CI, 6.8–7.6) deaths per 1000 patient‐years for those prescribed ≥50% of the dose (Table 3). The corresponding rates of MACE were 130.4 (95% CI, 128.7–132.1) and 73.0 (95% CI, 71.8–74.2) events per 1000 patient‐years, respectively. Similar patterns were observed in the HF cohort but with even more pronounced differences between patients on <50% and ≥50% of guideline‐recommended RAASi dose: mortality rates were 141.7 (95% CI, 136.4–147.3) compared with 12.5 (95% CI, 10.9–14.4) deaths per 1000 patient‐years, respectively, and MACE rates were 290.4 (95% CI, 282.6–298.3) versus 148.5 (95% CI, 142.7–154.5) events per 1000 patient‐years, respectively.

**Table 3:** Incidence of Mortality and MACE Stratified by RAASi Dose Within the Interval When These Events Occurred

Cohort	Outcome	RAASi Dose (of ESC Guideline‐Recommended)	Event Count	Rate Per 1000 Patient‐Years (95% CI)	Adjusted IRR (95% CI)
CKD	Mortality	<50%	10 506	57.73 (56.63–58.85)	5.59 (5.28–5.92)
		≥50%	1377	7.17 (6.80–7.56)	Reference
	MACE	<50%	23 726	130.38 (128.72–132.05)	1.61 (1.56–1.66)
		≥50%	14 004	72.95 (71.75–74.17)	Reference
HF	Mortality	<50%	2601	141.74 (136.35–147.30)	7.33 (6.34–8.47)
		≥50%	206	12.53 (10.87–14.36)	Reference
	MACE	<50%	5328	290.35 (282.61–298.26)	1.86 (1.72–2.00)
		≥50%	2442	148.49 (142.66–154.50)	Reference

CKD, chronic kidney disease; ESC, European Society of Cardiology; HF, heart failure; IRR, incident rate ratio; MACE, major adverse cardiac event; RAASi, renin–angiotensin–aldosterone system inhibitor.

After adjusting for covariates, statistically significant inverse associations were estimated between mean RAASi dose and the incidence of mortality and MACE (Table 3). In the CKD cohort, adjusted IRRs comparing patients on <50% of the recommended dose with those on >50% were 5.6 (95% CI, 5.3–5.9) for mortality and 1.6 (95% CI, 1.6–1.7) for MACE. In the HF cohort, larger adjusted IRRs of 7.3 (95% CI, 6.3–8.5) for mortality and 1.8 (95% CI, 1.7–2.0) for MACE were estimated.

## Discussion

This study investigated the associations between (1) hyperkalemia (a potential adverse effect of RAASi treatment) and dosing of RAASi in CKD and HF patients in the United Kingdom and (2) suboptimal RAASi dosing and adverse clinical outcomes, including all‐cause mortality and nonfatal MACE.

RAASi dosing during the study follow‐up period was frequently suboptimal when compared with the ESC guidelines,^3^ with 41.8% of prescriptions for CKD patients and 36.5% for HF patients including less than half of the guideline‐recommended dose. This was a particular concern for ARBs and ACEIs, whereas prescribers appeared to be more adherent to the ESC guidelines regarding MRA doses.

RAASi dose down‐titrations and discontinuations during the study follow‐up period were rare, although the odds of discontinuation and down‐titration were higher in patients with hyperkalemia than those without. This became more prominent as the K^+^ threshold used to define hyperkalemia increased.

Over 10 years, cumulative incidence of death was consistently highest in both CKD and HF patients receiving <50% of the ESC guideline‐recommended dose, followed by those receiving no RAASi, and was lowest for patients who spent most of their follow‐up on ≥50% of the ESC‐recommended dose. The cumulative incidence of nonfatal MACE, however, showed a different pattern from mortality in that it was lowest in both CKD and HF patients receiving no RAASi, followed by patients receiving ≥50% of the recommended dose and highest in patients receiving <50% of the recommended dose. A possible explanation for this finding is that the use of RAASi reduces the severity of MACE events, so the majority of MACE in non‐RAASi patients would be fatal, and this study considered only nonfatal MACE. RAASi use has been associated with lower severity of some MACE events, including myocardial infarction^30^ and stroke,^31^, ^32^ although the evidence for the latter appears inconclusive.^33^ The hypothesis that patients not in receipt of RAASi are more likely to experience severe, fatal MACE is plausible given the excess mortality risk observed in these patients in the present study. However, it is also possible that the non‐RAASi cohort includes a mixture of patients (1) who are well‐managed on other medications; (2) who have HF with preserved ejection fraction, which has no approved therapy that has been demonstrated to reduce mortality and thus may have no firm indication for RAASi treatment; and (3) who are at high risk of hyperkalemia‐related complications so that RAASi is contraindicated. The potential heterogeneity of this group warrants caution when interpreting the results observed in these patients. Independent of RAASi dose received, recent history of MACE significantly increased the incidence of mortality in both the CKD and HF cohorts (Tables S4 and S5, respectively).

Among patients receiving RAASi, doses >50% of the ESC guideline‐recommended dose were associated with a consistently lower cumulative incidence of mortality and MACE compared with doses <50%, supporting the beneficial effects of adherence to prescribing guidelines concerning dosing. Indeed, in the CKD cohort, suboptimal RAASi dosing within a given quarter was associated with significantly higher odds of death and MACE occurring within the same quarter, although the association was more pronounced for death than MACE (IRR: 5.60 and 1.60, respectively). HF patients receiving suboptimal RAASi dosing in a given quarter were at 7.34 times higher risk of imminent death than those receiving ≥50% of the recommended dose, but the relationship was, again, less prominent for MACE (IRR: 1.85). This result may indicate that RAASi activation is a more important driver of mortality in HF patients than in CKD patients.

We aimed to expand on previous studies, which have investigated either associations between K^+^ levels and RAASi dosing or RAASi dosing and patient outcomes,^16^, ^17^, ^18^, ^20^ providing a fuller picture of the real‐world links among hyperkalemia, RAASi dose, and adverse clinical outcomes. Earlier studies typically featured relatively small patient samples, short follow‐up periods, and lack of adjustment for patient and disease characteristics that could confound the relationship of RAASi dose, serum K^+^, and patient outcomes. In contrast, this study included a large cohort of UK patients with new‐onset CKD or HF, provided up to 10 years of follow‐up, and attempted to minimize bias by adjusting for differences in patient clinical and demographic characteristics.

Limitations of the current study arise mainly from its retrospective design. Primarily, the study was able to determine only associations rather than causality between hyperkalemia and changes to RAASi dosing and between RAASi dosing and adverse clinical outcomes. To focus more clearly on discontinuation or down‐titration of RAASi associated with hyperkalemia, we excluded from the analysis any RAASi prescriptions issued after a renal failure event, which included both acute kidney injury (that may potentially be caused or exacerbated by RAASi use^29^) and end‐stage renal disease. Many covariates available within the CPRD are not routinely collected in National Health Service primary care, which leads to few patients having a complete set of covariates, reflecting the time and organizational constraints imposed on primary care in England. The proportion of missing data is consistent with other publications utilizing the CPRD database^34^, ^35^, ^36^ and therefore can be considered to reflect the patterns of testing in routine primary care. Missing data were filled in using multiple imputation methods that preserve its inherent variability and uncertainty and that are frequently used in research based on clinical databases.^37^, ^38^ This approach was favored over including only patients who had a complete set of covariates in the analysis, which would produce a greatly reduced data set and result in bias within the analyses.

Despite the employment of analytical and statistical measures to control for clinically relevant covariates, it is possible that additional factors that were not assessed in this study could have contributed to the observed associations, particularly when considering MACE and mortality, which are likely to arise from multiple causes that may not always be possible to define clearly. Furthermore, there are no published guidelines regarding RAASi dosing in patients with CKD, so recommended therapies and doses were assumed to be the same as those for HF patients. Finally, although the CPRD linked to HES data includes information on primary and secondary care provided to a large and diverse sample of patients, the use of these linked databases as the sole data source renders the study reliant on the accuracy and completeness of CPRD and HES data entry and restricts the population to the UK setting.

Despite the aforementioned limitations, this study provides important real‐world data on the characteristics of CKD and HF patients treated with RAASi in the United Kingdom and on the associations of RAASi dose, hyperkalemia, and adverse clinical outcomes. Underprescribing of RAASi was ubiquitous among the study population and increased following hyperkalemia events. The results highlight the potential negative impact of suboptimal RAASi dosing, indicate the generalizability of ESC‐recommended RAASi doses in HF to CKD patients, and emphasize the need for strategies that allow patients to be maintained on appropriate therapy, avoiding RAASi dose modification or discontinuation.

## Sources of Funding

This study was funded by AstraZeneca. The funding agreement ensured the authors’ independence in designing the study, interpreting the data, and preparing the article for publication.

## Disclosures

Linde received significant institutional research grant funding from AstraZeneca and modest speaker honoraria from Medtronic, Vifor, Bayer, Impulse Dynamics, and Abbot. Bakhai received modest advisory honoraria from AstraZeneca in relation to this study. Furuland received modest advisory honoraria from AstraZeneca and Boehringer‐Ingelheim. Evans received significant funding from Novo Nordisk, Novartis, and Boehringer‐Ingelheim. McEwan and Ayoubkhani received significant research grant funding from AstraZeneca in relation to this study. Qin received significant funding from AstraZeneca as a full‐time employee and stock holder.

## Supporting information


**Data S1.** Supplementary methods.
**Table S1.** Proportion of Missing Data at Baseline for Continuous Variables in the Chronic Kidney Disease and Heart Failure Cohorts
**Table S2.** Model Output for Dose Modification of Renin–Angiotensin–Aldosterone System Inhibitors, Stratified by Serum Potassium Threshold for the Chronic Kidney Disease Cohort
**Table S3.** Model Output for Dose Modification of Renin–Angiotensin–Aldosterone System Inhibitors, Stratified by Serum Potassium Threshold for the Heart Failure Cohort
**Table S4.** Model Output for Adverse Outcomes Stratified by Renin–Angiotensin–Aldosterone System Inhibitors Dose Within Interval Outcome Occurred for the Chronic Kidney Disease Cohort
**Table S5.** Model Output for Adverse Outcomes Stratified by Renin–Angiotensin–Aldosterone System Inhibitors Dose Within Interval Outcome Occurred for the Heart Failure Cohort
**Table S6.** Model Output for Survival Analysis of Adverse Outcomes Stratified by Majority Renin–Angiotensin–Aldosterone System Inhibitors Dose Over the Follow‐Up for the Chronic Kidney Disease Cohort
**Table S7.** Model Output for Survival Analysis of Adverse Outcomes Stratified by Majority Achieved Renin–Angiotensin–Aldosterone System Inhibitors Dose Over the Follow‐Up for the Heart Failure Cohort
**Table S8.** Summary of Sample Size and Patient Attrition
**Table S9.** Baseline Patient Demographics, Clinical Characteristics, and Clinical Histories of Chronic Kidney Disease and Heart Failure Patients Included in the Analysis (Renin–Angiotensin–Aldosterone System Inhibitors [RAASi]) and Patients Excluded Due to Receiving RAASi Agents for Which a Recommended Dose was Not Specified by the European Society of Cardiology Guidelines^1^ or Due to Missing or Unusable RAASi Dose Information
**Figure S1.** Illustrative example of data structuring for estimating associations between renin–angiotensin–aldosterone system inhibitor dose and adverse clinical outcomes.
**Figure S2.** Three stages of the multiple imputation process.
**Figure S3.** Simple description of the chained equations algorithm.

## References

[1] Atlas SA . The renin‐angiotensin aldosterone system: pathophysiological role and pharmacologic inhibition. J Manag Care Pharm. 2007;13:9–20. 17970613 10.18553/jmcp.2007.13.s8-b.9 PMC10437584

[2] Ferrario CM , Mullick AE . Renin angiotensin aldosterone inhibition in the treatment of cardiovascular disease. Pharmacol Res. 2017;125:57–71. 28571891 10.1016/j.phrs.2017.05.020 PMC5648016

[3] Ponikowski P , Voors AA , Anker SD , Bueno H , Cleland JGF , Coats AJS , Falk V , Gonzalez‐Juanatey JR , Harjola VP , Jankowska EA , Jessup M , Linde C , Nihoyannopoulos P , Parissis JT , Pieske B , Riley JP , Rosano GMC , Ruilope LM , Ruschitzka F , Rutten FH , van der Meer P ; ESC Scientific Document Group . 2016 ESC guidelines for the diagnosis and treatment of acute and chronic heart failure: the task force for the diagnosis and treatment of acute and chronic heart failure of the European Society of Cardiology (ESC). Developed with the special contribution of the Heart Failure Association (HFA) of the ESC. Eur Heart J. 2016;37:2129–2200. 27206819 10.1093/eurheartj/ehw128

[4] National Institute for Health and Care Excellence . Chronic heart failure in adults: management [CG108]. August 2010 Available at: https://www.nice.org.uk/guidance/cg108/. Accessed June 29, 2018. 30645061

[5] Becker GJ , Wheeler DC , De Zeeuw D , Fujita T , Furth SL , Holdaas H , Mendis S , Oparil S , Perkovic V , Rodrigues CIS , Sarnak MJ , Schernthaner G , Tomson CRV , Zoccali C . KDIGO clinical practice guideline for the management of blood pressure in chronic kidney disease. Kidney Int Suppl. 2012;2:337–414.

[6] National Institute for Health and Care Excellence . Chronic kidney disease in adults: assessment and management [CG182]. January 2015 Available at: https://www.nice.org.uk/guidance/cg182/chapter/1-Recommendations#classification-of-chronic-kidney-disease-2. Accessed July 2, 2018. 32208570

[7] National Institute for Health and Care Excellence . Hypertension in adults: diagnosis and management [CG127]. November 2016 Available at: https://www.nice.org.uk/guidance/cg127. Accessed July 2, 2018.

[8] Pechlivanidis G , Mantziari L , Giannakoulas G , Dimitroula H , Styliadis H , Karvounis H , Styliadis IH , Parharidis G . Effects of renin‐angiotensin system inhibition on right ventricular function in patients with mild essential hypertension. J Renin Angiotensin Aldosterone Syst. 2011;12:358–364. 21436206 10.1177/1470320310391334

[9] Epstein M . Hyperkalemia as a constraint to therapy with combination renin‐angiotensin system blockade: the elephant in the room. J Clin Hypertens (Greenwich). 2009;11:55–60. 19222668 10.1111/j.1751-7176.2008.00071.x PMC8673296

[10] Raebel MA . Hyperkalemia associated with use of angiotensin‐converting enzyme inhibitors and angiotensin receptor blockers. Cardiovasc Ther. 2012;30:e156–e166. 21883995 10.1111/j.1755-5922.2010.00258.x

[11] Viera AJ , Wouk N . Potassium disorders: hypokalemia and hyperkalemia. Am Fam Physician. 2015;92:487–495. 26371733

[12] Esposito C , Bellotti N , Fasoli G , Foschi A , Plati AR , Dal Canton A . Hyperkalemia‐induced ECG abnormalities in patients with reduced renal function. Clin Nephrol. 2004;62:465–468. 15630907 10.5414/cnp62465

[13] Collins AJ , Pitt B , Reaven N , Funk S , McGaughey K , Wilson D , Bushinsky DA . Association of serum potassium with all‐cause mortality in patients with and without heart failure, chronic kidney disease, and/or diabetes. Am J Nephrol. 2017;46:213–221. 28866674 10.1159/000479802 PMC5637309

[14] Nakhoul GN , Huang H , Arrigain S , Jolly SE , Schold JD , Nally JV Jr , Navaneethan SD . Serum potassium, end‐stage renal disease and mortality in chronic kidney disease. Am J Nephrol. 2015;41:456–463. 26228532 10.1159/000437151 PMC4686260

[15] Alfonzo A , Soar J , MacTier R , Fox J , Shillday I , Nolan J , Kishen R , Douglas A , Bartlett B , Wiese M , Wilson B , Beatson J , Allen L , Goolam M , Whittle M . Treatment of acute hyperkalaemia in adults. March 2014 Available at: https://renal.org/wp-content/uploads/2017/06/hyperkalaemia-guideline-1.pdf and https://renal.org/wp-content/uploads/2017/10/HYPERKALAEMIA-ALGORITHM-MARCH-2014.pdf. Accessed July 2, 2018.

[16] Komajda M , Cowie MR , Tavazzi L , Ponikowski P , Anker SD , Filippatos GS ; QUALIFY Investigators . Physicians’ guideline adherence is associated with better prognosis in outpatients with heart failure with reduced ejection fraction: the QUALIFY international registry. Eur J Heart Fail. 2017;19:1414–1423. 28463464 10.1002/ejhf.887

[17] Maggioni AP , Anker SD , Dahlstrom U , Filippatos G , Ponikowski P , Zannad F , Amir O , Chioncel O , Leiro MC , Drozdz J , Erglis A , Fazlibegovic E , Fonseca C , Fruhwald F , Gatzov P , Goncalvesova E , Hassanein M , Hradec J , Kavoliuniene A , Lainscak M , Logeart D , Merkely B , Metra M , Persson H , Seferovic P , Temizhan A , Tousoulis D , Tavazzi L ; Heart Failure Association of the ESC . Are hospitalized or ambulatory patients with heart failure treated in accordance with European Society of Cardiology guidelines? Evidence from 12,440 patients of the ESC Heart Failure Long‐Term Registry. Eur J Heart Fail. 2013;15:1173–1184. 23978433 10.1093/eurjhf/hft134

[18] Ouwerkerk W , Voors AA , Anker SD , Cleland JG , Dickstein K , Filippatos G , van der Harst P , Hillege HL , Lang CC , Ter Maaten JM , Ng LL , Ponikowski P , Samani NJ , van Veldhuisen DJ , Zannad F , Metra M , Zwinderman AH . Determinants and clinical outcome of uptitration of ACE‐inhibitors and beta‐blockers in patients with heart failure: a prospective European study. Eur Heart J. 2017;38:1883–1890. 28329163 10.1093/eurheartj/ehx026

[19] Epstein M , Reaven NL , Funk SE , McGaughey KJ , Oestreicher N , Knispel J . Evaluation of the treatment gap between clinical guidelines and the utilization of renin‐angiotensin‐aldosterone system inhibitors. Am J Manag Care. 2015;21:S212–S220. 26619183

[20] Schmidt M , Mansfield KE , Bhaskaran K , Nitsch D , Sorensen HT , Smeeth L , Tomlinson LA . Adherence to guidelines for creatinine and potassium monitoring and discontinuation following renin‐angiotensin system blockade: a UK general practice‐based cohort study. BMJ Open. 2017;7:e012818. 10.1136/bmjopen-2016-012818 PMC5223644 28069618

[21] CPRD UK data driving real‐world evidence. Data. September 21, 2018 Available from: https://cprd.com/Data. Accessed October 18, 2018.

[22] CPRD UK data driving real‐world evidence. CPRD linked data. August 16, 2018 Available from: https://cprd.com/linked-data. Accessed October 18, 2018.

[23] Luo J , Brunelli SM , Jensen DE , Yang A . Association between serum potassium and outcomes in patients with reduced kidney function. Clin J Am Soc Nephrol. 2016;11:90–100. 26500246 10.2215/CJN.01730215 PMC4702219

[24] Furuland H , McEwan P , Evans M , Linde C , Ayoubkhani D , Bakhai A , Palaka E , Bennett H , Qin L . Serum potassium as a predictor of adverse clinical outcomes in patients with chronic kidney disease: new risk equations using the UK Clinical Practice Research Datalink. BMC Nephrol. 2018;19:211. 30134846 10.1186/s12882-018-1007-1 PMC6106824

[25] Azur MJ , Stuart EA , Frangakis C , Leaf PJ . Multiple imputation by chained equations: what is it and how does it work? Int J Methods Psychiatr Res. 2011;20:40–49. 21499542 10.1002/mpr.329 PMC3074241

[26] van Buuren S , Groothuis‐Oudshoorn K . Mice: multivariate imputation by chained equations in R. J Stat Softw. 2011 Available at: https://www.jstatsoft.org/issue/view/v045. DOI: 10.18637/jss.v045.i03.

[27] Rubin DB . Multiple Imputation for Nonresponse in Surveys. New York, NY: John Wiley and Sons Ltd; 2004.

[28] R Core Team . R: a language and environment for statistical computing. Available at: https://www.r-project.org/. Accessed September 6, 2018.

[29] Tomson C , Tomlinson LA . Stopping RAS inhibitors to minimize AKI. More harm than good? Clin J Am Soc Nephrol. 2019;14:617–619. 30814113 10.2215/CJN.14021118 PMC6450359

[30] Li M , Huang Y , Du X , Li S , Ji J , Patel A , Gao R , Wu Y . Impact of prior use of four preventive medications on outcomes in patients hospitalized for acute coronary syndrome‐results from CPACS‐2 study. PLoS One. 2016;11:e0163068. 27626640 10.1371/journal.pone.0163068 PMC5023149

[31] Miyamoto N , Tanaka Y , Ueno Y , Tanaka R , Hattori N , Urabe T . Benefits of prestroke use of angiotensin type 1 receptor blockers on ischemic stroke severity. J Stroke Cerebrovasc Dis. 2012;21:363–368. 21094055 10.1016/j.jstrokecerebrovasdis.2010.09.011

[32] Selim M , Savitz S , Linfante I , Caplan L , Schlaug G . Effect of pre‐stroke use of ACE inhibitors on ischemic stroke severity. BMC Neurol. 2005;5:10. 15949043 10.1186/1471-2377-5-10 PMC1175849

[33] Desmaele S , Cornu P , Barbe K , Brouns R , Steurbaut S , Dupont AG . Relationship between pre‐stroke cardiovascular medication use and stroke severity. Eur J Clin Pharmacol. 2016;72:495–502. 26706251 10.1007/s00228-015-2001-1

[34] Hippisley‐Cox J , Coupland C , Brindle P . The performance of seven QPrediction risk scores in an independent external sample of patients from general practice: a validation study. BMJ Open. 2014;4:e005809. 10.1136/bmjopen-2014-005809 PMC4156807 25168040

[35] Booth H , Kahn O , Prevost T , Reddy M , Dregan A , Charlton J , Ashworth M , Rudisill C , Littlejohns P , Gulliford MC . Incidence of type 2 diabetes after bariatric surgery: population‐based matched cohort study. Lancet Diabetes Endocrinol. 2014;2:963–968. 25466723 10.1016/S2213-8587(14)70214-1

[36] Bhaskaran K , Forbes HJ , Douglas I , Leon DA , Smeeth L . Representativeness and optimal use of body mass index (BMI) in the UK Clinical Practice Research Datalink (CPRD). BMJ Open. 2013;3:e003389. 10.1136/bmjopen-2013-003389 PMC3773634 24038008

[37] Marston L , Carpenter JR , Walters KR , Morris RW , Nazareth I , Petersen I . Issues in multiple imputation of missing data for large general practice clinical databases. Pharmacoepidemiol Drug Saf. 2010;19:618–626. 20306452 10.1002/pds.1934

[38] Sterne JA , White IR , Carlin JB , Spratt M , Royston P , Kenward MG , Wood AM , Carpenter JR . Multiple imputation for missing data in epidemiological and clinical research: potential and pitfalls. BMJ. 2009;338:b2393. 19564179 10.1136/bmj.b2393 PMC2714692

