# Supplementary material for: Real‐World Associations of Renin–Angiotensin–Aldosterone System Inhibitor Dose, Hyperkalemia, and Adverse Clinical Outcomes in a Cohort of Patients With New‐Onset Chronic Kidney Disease or Heart Failure in the United Kingdom
Source: J Am Heart Assoc. 2019 Nov 12;8(22):e012655. doi: 10.1161/JAHA.119.012655 (PMC6915283; doi:10.1161/JAHA.119.012655)
Supplement: Supplementary file 1 — Data S1. Supplementary methods. Table S1. Proportion of Missing Data at Baseline for Continuous Variables in the Chronic Kidney Disease and Heart Failure Cohorts Table S2. Model Output for Dose Modification of Renin–Angiotensin–Aldosterone System Inhibitors, Stratified by Serum Potassium Threshold for the Chronic Kidney Disease Cohort Table S3. Model Output for Dose Modification of Renin–Angiotensin–Aldosterone System Inhibitors, Stratified by Serum Potassium Threshold for the Heart Failure Cohort Table S4. Model Output for Adverse Outcomes Stratified by Renin–Angiotensin–Aldosterone System Inhibitors Dose Within Interval Outcome Occurred for the Chronic Kidney Disease Cohort Table S5. Model Output for Adverse Outcomes Stratified by Renin–Angiotensin–Aldosterone System Inhibitors Dose Within Interval Outcome Occurred for the Heart Failure Cohort Table S6. Model Output for Survival Analysis of Adverse Outcomes Stratified by Majority Renin–Angiotensin–Aldosterone System Inhibitors Dose Over the Follow‐Up for the Chronic Kidney Disease Cohort Table S7. Model Output for Survival Analysis of Adverse Outcomes Stratified by Majority Achieved Renin–Angiotensin–Aldosterone System Inhibitors Dose Over the Follow‐Up for the Heart Failure Cohort Table S8. Summary of Sample Size and Patient Attrition Table S9. Baseline Patient Demographics, Clinical Characteristics, and Clinical Histories of Chronic Kidney Disease and Heart Failure Patients Included in the Analysis (Renin–Angiotensin–Aldosterone System Inhibitors [RAASi]) and Patients Excluded Due to Receiving RAASi Agents for Which a Recommended Dose was Not Specified by the European Society of Cardiology Guidelines1 or Due to Missing or Unusable RAASi Dose Information Figure S1. Illustrative example of data structuring for estimating associations between renin–angiotensin–aldosterone system inhibitor dose and adverse clinical outcomes. Figure S2. Three stages of the multiple imputation process. Figure S3. Simple description of [file JAH3-8-e012655-s001.pdf]

Supplemental Material

Data S1.

Supplementary Methods

Calculation of mean RAASi doses

Each patient's follow-up time was divided into quarters, each consisting of 91.3125 days, and only patient-quarters spent on RAASi were included in the analysis, while intervals of discontinuation were omitted. Mean RAASi dose within each quarter (expressed as percentage of the ESC guideline-recommended dose) was calculated as the mean dose (also expressed as percentage of the ESC guideline-recommended dose for each therapy) across all therapies within each quarter, weighted by proportion of the quarter that the patient was on each therapy. Where a therapy was discontinued during a quarter, a dose of 0 was assumed for that therapy from the date of discontinuation to the end of the interval and included in the quarterly average. Specifically, we compared a patient's dosage within each quarter to the ESC 2016 guidelines [1], as well as partly using each patient's history of RAASi therapy. If a patient had never been prescribed an MRA, it was assumed that MRA treatment was not required. Once a patient was prescribed an MRA, MRA treatment was assumed to be required from then on and treatment discontinuations were considered as described in the manuscript for all other RAASi therapies. The following summarises ESC guidelines on RAASi treatment, as used for the purpose of this analysis:

- ACE inhibitors are recommended in all symptomatic patients with HF with reduced left-ventricular ejection fraction (LVEF), unless contraindicated or not tolerated

- ARBs are recommended only as an alternative in patients intolerant of ACE inhibitors
- MRAs are recommended in all patients symptomatic despite treatment with an ACE inhibitor, who have heart failure with reduced LVEF and their LVEF is ≤35%

In the absence of LVEF data, total target dose prior to initiation of MRA therapy was assumed to be 100% of an ACEi/ARB dose only (i.e. MRA was assumed not to be required). For all periods after initiation on MRA therapy, total target dose was assumed to be 100% of an ACEi/ARB dose + 100% of an MRA dose (i.e. MRA was assumed to be required from this point onwards). An example calculation of the total RAASi dose as a percentage of the guideline-recommended dose is presented in Figure S1. Note, that despite the ESC guidelines recommending ARBs as an alternative to ACE inhibitors in case of intolerance, patients receiving ACE inhibitors and ARBs concomitantly were not excluded from the analyses.

Missing data: Multiple imputation methods

Final estimates of adjusted incidence rate ratios (IRRs) and hazard ratios (HRs) were derived from five multiply imputed datasets, as illustrated in Figure S2. The model coefficients and their standard errors from each of the five imputed datasets were pooled to produce the final set of estimates using Rubin Rules, as described by Carpenter et al[2]. All missing baseline values of clinical variables (where patients did not have a measurement taken at index) were estimated using multiple imputation, with the last observation carried forward (LOCF) method then being applied for time periods between clinical measurements. The application of multiple imputation to

impute missing values in datasets derived from large clinical databases has been explored in other studies and has been shown to provide valid results[3, 4].
Multiple imputation was performed by the method of Chained Equations[5] as implemented in R package mice[6] (Figure S3).

For this study, k=5 completed datasets were produced after performing i=50 iterations for the HF and CKD cohorts (this was sufficient to ensure convergence of the imputations).

The default method used by 'mice' for step (4) in the above algorithm is predictive mean matching (PMM); however, for this study linear regression predictions were used directly due to the low percentage of complete data for some variables. The multiple imputation models included the full set of candidate covariates from the analysis models that were subsequently fitted to the imputed datasets, plus each patient's total follow-up time and their observed number of events (deaths, major adverse cardiovascular events [MACE] and RAASi discontinuation) over the study follow-up period.

All clinical variables except serum potassium and estimated glomerular filtration rate (eGFR) were log transformed prior to fitting imputation models to enforce normality of their distributions. For these variables, a retransformation bias-correction factor was applied to the imputations after they were back-transformed to their original scale.

Sample size and patient attrition

The study cohort was derived from all patients on the Clinical Practice Research Datalink (CPRD) (making use of linked Hospital Episode Statistics [HES] data) aged ≥18 years between 1 January 2006 and 31 December 2015. Patients not in receipt of renin-angiotensin-aldosterone system inhibitor (RAASi) therapies, defined as a composite of specific angiotensin-converting enzyme (ACE) inhibitors, angiotensin receptor blockers (ARBs) and mineralocorticoid receptor antagonists (MRAs) for which a recommended dose is provided by the European Society of Cardiology (ESC) 2016 Guidelines[1], at any point over the duration of the follow-up period were included in some analyses for comparative purposes. Patients who received ESC-recommended RAASi therapy but had no adequate information on treatment dose available were also excluded. Guideline-recommended therapies included 4 ACE-Is (ramipril, lisinopril, enalapril maleate, captopril), 3 ARBs (candesartan cilexetil, losartan potassium and valsartan) and 2 MRAs (spironolactone and eplerenone). Specific inclusion and exclusion criteria for chronic kidney disease (CKD) and heart failure (HF) patients are listed in the manuscript. Table S8 provides details of sample size and patient attrition.

Table S1. Proportion of missing data at baseline for continuous variables in the CKD and HF cohorts.

Variable	CKD cohort			HF cohort		
	RAASi dose achieved during the majority (≥75%) of follow-up			RAASi dose achieved during the majority (≥75%) of follow-up		
	Non-RAASi (n=71,008)	<50% of ESC-recommended dose (n=27,935)†	≥50% of ESC-recommended dose (n=26,596)†	Non-RAASi (n=6,063)	<50% of ESC-recommended dose (n=4,568)†	≥50% of ESC-recommended dose (n=2,758)†
**Baseline* patient demographics and clinical characteristics**						
BMI (kg/m^2)	44,857 (63.16%)	13,504 (48.34%)	11,164 (41.98%)	5,361 (88.41%)	2,515 (55.06%)	1,056 (38.29%)
SBP (mmHg)	20,105 (28.31%)	1,812 (6.49%)	850 (3.20%)	4,262 (70.28%)	609 (13.33%)	123 (4.46%)
DBP (mmHg)	20,105 (28.31%)	1,812 (6.49%)	850 (3.20%)	4,262 (70.28%)	609 (13.33%)	123 (4.46%)
eGFR (mL/min/1.73m2)	25,681 (36.16%)	11,398 (40.80%)	9,746 (36.64%)	5,558 (91.66%)	2,946 (64.49%)	1,666 (60.41%)
Serum potassium (mEq/L)	11,427 (16.09%)	2,478 (8.87%)	1,796 (6.75%)	4,625 (76.27%)	909 (19.90%)	310 (11.24%)
Serum phosphorus (mEq/L)	54,739 (77.08%)	22,127 (79.21%)	21,583 (81.15%)	5,744 (94.72%)	3,869 (84.70%)	2,330 (84.48%)
Total Cholesterol (mmol/L)	34,271 (48.26%)	8,964 (32.09%)	6,302 (23.70%)	5,347 (88.18%)	2,383 (52.17%)	896 (32.49%)
WBC	22,206 (31.27%)	9,234 (33.06%)	9,323 (35.05%)	4,818 (79.45%)	1,744 (38.18%)	961 (34.84%)

BMI: body mass index; CKD: chronic kidney disease; DBP: diastolic blood pressure; eGFR: estimated glomerular filtration rate; HF: heart failure; RAASi: renin-angiotensin-aldosterone system inhibitors; SBP: systolic blood pressure; WBC: white blood cell count.

*Baseline for RAASi patients is time of each patient's first RAASi prescription after their first CKD/HF event, for non-RAASi patients is time of first CKD/HF event. †The numbers of patients receiving <50% and ≥50% of the recommended dose do not add up to the total cohort size because patients who spent most of their time on 0% dose and those who do not have a clear majority of time spent at a given dose level are not shown in the Table but are included in the total cohort

Table S2. Model output for dose modification of RAASi, stratified by serum K+ threshold for CKD cohort.

Explanatory variable	Outcome	OR	95% lower CI	95% upper CI	P-value
**Incidence of down-titration or discontinuation: Hyperkalaemia threshold: 5.0 mmol/L**					
Serum potassium: ≥5.0 mmol/L	Down-titration	1.7941	1.6449	1.9568	<.0001
Serum potassium: ≥5.0 mmol/L	Discontinuation	1.2707	1.1710	1.3790	<.0001
Drug type: ARB	Down-titration	0.8142	0.6857	0.9667	0.0189
Drug type: ARB	Discontinuation	1.0566	0.9324	1.1974	0.3874
Drug type: MRA	Down-titration	1.1947	1.0887	1.3110	0.0002
Drug type: MRA	Discontinuation	0.6329	0.5772	0.6940	<.0001
Age (years)	Down-titration	1.0074	1.0031	1.0118	0.0009
Age (years)	Discontinuation	1.0042	1.0003	1.0082	0.0332
Gender at baseline: Male	Down-titration	1.1268	1.0370	1.2245	0.0049
Gender at baseline: Male	Discontinuation	1.0573	0.9822	1.1381	0.1381
Time from baseline (years)	Down-titration	0.9954	0.9790	1.0121	0.5895
Time from baseline (years)	Discontinuation	1.0036	0.9886	1.0189	0.643
Time-updated eGFR: < 15 mL/min/1.73m^2	Down-titration	0.5506	0.2911	1.0415	0.0666
Time-updated eGFR: < 15 mL/min/1.73m^2	Discontinuation	1.6700	1.1067	2.5199	0.0146
Time-updated eGFR: 30 to <45 mL/min/1.73m^2	Down-titration	0.5268	0.4402	0.6304	<.0001
Time-updated eGFR: 30 to <45 mL/min/1.73m^2	Discontinuation	0.5178	0.4433	0.6049	<.0001
Time-updated eGFR: 45 to <60 mL/min/1.73m^2	Down-titration	0.5213	0.4488	0.6055	<.0001
Time-updated eGFR: 45 to <60 mL/min/1.73m^2	Discontinuation	0.4976	0.4362	0.5676	<.0001
Time-updated eGFR: ≥ 60 mL/min/1.73m^2	Down-titration	0.7130	0.6101	0.8332	<.0001
Time-updated eGFR: ≥ 60 mL/min/1.73m^2	Discontinuation	0.6516	0.5672	0.7486	<.0001
Time-updated blood pressure: Low	Down-titration	1.9265	1.7171	2.1614	<.0001
Time-updated blood pressure: Low	Discontinuation	1.2531	1.1217	1.3998	<.0001
Time-updated blood pressure: Normal	Down-titration	1.3356	1.2190	1.4634	<.0001
Time-updated blood pressure: Normal	Discontinuation	1.3454	1.2442	1.4549	<.0001
Time-updated blood pressure: Pre-High	Down-titration	5.6593	3.8225	8.3787	<.0001
Time-updated blood pressure: Pre-High	Discontinuation	1.9398	1.1596	3.2450	0.0116
Concomitant diuretics: Yes	Down-titration	0.7996	0.1101	5.8051	0.8251
Concomitant diuretics: Yes	Discontinuation	0.5597	0.0748	4.1901	0.572
Concomitant NSAIDS: Yes	Down-titration	0.0007	0.0004	0.0014	<.0001
Concomitant NSAIDS: Yes	Discontinuation	2.4010	0.2704	21.3177	0.4318
Concomitant Betablockers: Yes	Down-titration	1.8414	0.5549	6.1106	0.3184
Concomitant Betablockers: Yes	Discontinuation	0.9140	0.2134	3.9158	0.9037
Concomitant RAASi: Yes	Down-titration	1.4096	1.2434	1.5980	<.0001
Concomitant RAASi: Yes	Discontinuation	3.0102	2.7026	3.3528	<.0001
**Incidence of down-titration or discontinuation: Hyperkalaemia threshold: 5.5 mmol/L**					
Serum potassium: ≥5.5 mmol/L	Down-titration	2.9523	2.6222	3.3240	<.0001
Serum potassium: ≥5.5 mmol/L	Discontinuation	1.8004	1.5937	2.0338	<.0001
Drug type: ARB	Down-titration	0.8082	0.6808	0.9593	0.0149
Drug type: ARB	Discontinuation	1.0540	0.9301	1.1944	0.41
Drug type: MRA	Down-titration	1.1910	1.0851	1.3072	0.0002
Drug type: MRA	Discontinuation	0.6332	0.5775	0.6943	<.0001
Age (years)	Down-titration	1.0077	1.0034	1.0121	0.0005
Age (years)	Discontinuation	1.0044	1.0005	1.0084	0.0266
Gender at baseline: Male	Down-titration	1.1277	1.0376	1.2257	0.0047
Gender at baseline: Male	Discontinuation	1.0559	0.9809	1.1367	0.1485
Time from baseline (years)	Down-titration	0.9961	0.9795	1.0130	0.6481
Time from baseline (years)	Discontinuation	1.0037	0.9887	1.0190	0.6358
Time-updated eGFR: < 15 mL/min/1.73m^2	Down-titration	0.5002	0.2622	0.9542	0.0355
Time-updated eGFR: < 15 mL/min/1.73m^2	Discontinuation	1.5831	1.0383	2.4138	0.0328
Time-updated eGFR: 30 to <45 mL/min/1.73m^2	Down-titration	0.5438	0.4535	0.6522	<.0001
Time-updated eGFR: 30 to <45 mL/min/1.73m^2	Discontinuation	0.5325	0.4555	0.6226	<.0001
Time-updated eGFR: 45 to <60 mL/min/1.73m^2	Down-titration	0.5409	0.4642	0.6303	<.0001
Time-updated eGFR: 45 to <60 mL/min/1.73m^2	Discontinuation	0.5114	0.4478	0.5841	<.0001
Time-updated eGFR: ≥ 60 mL/min/1.73m^2	Down-titration	0.7370	0.6293	0.8631	0.0002

Time-updated eGFR: ≥ 60 mL/min/1.73m^2	Discontinuation	0.6656	0.5788	0.7655	<.0001
Time-updated blood pressure: Low	Down-titration	1.9278	1.7180	2.1633	<.0001
Time-updated blood pressure: Low	Discontinuation	1.2557	1.1239	1.4030	<.0001
Time-updated blood pressure: Normal	Down-titration	1.3324	1.2159	1.4601	<.0001
Time-updated blood pressure: Normal	Discontinuation	1.3466	1.2453	1.4562	<.0001
Time-updated blood pressure: Pre-High	Down-titration	5.4882	3.6607	8.2279	<.0001
Time-updated blood pressure: Pre-High	Discontinuation	1.9224	1.1524	3.2071	0.0123
Concomitant diuretics: Yes	Down-titration	0.7749	0.1056	5.6878	0.802
Concomitant diuretics: Yes	Discontinuation	0.5306	0.0687	4.0987	0.5434
Concomitant NSAIDS: Yes	Down-titration	0.0007	0.0004	0.0013	<.0001
Concomitant NSAIDS: Yes	Discontinuation	2.3570	0.2798	19.8521	0.4303
Concomitant Betablockers: Yes	Down-titration	2.0334	0.6458	6.4025	0.2253
Concomitant Betablockers: Yes	Discontinuation	0.9589	0.2238	4.1087	0.9549
Concomitant RAASi: Yes	Down-titration	1.3978	1.2325	1.5852	<.0001
Concomitant RAASi: Yes	Discontinuation	2.9892	2.6832	3.3301	<.0001
**Incidence of down-titration or discontinuation: Hyperkalaemia threshold: 6.0 mmol/L**					
Serum potassium: ≥6.0 mmol/L	Down-titration	4.3185	3.5042	5.3219	<.0001
Serum potassium: ≥6.0 mmol/L	Discontinuation	2.8982	2.3343	3.5984	<.0001
Drug type: ARB	Down-titration	0.8069	0.6802	0.9573	0.0139
Drug type: ARB	Discontinuation	1.0536	0.9297	1.1939	0.4135
Drug type: MRA	Down-titration	1.1816	1.0766	1.2969	0.0004
Drug type: MRA	Discontinuation	0.6316	0.5760	0.6925	<.0001
Age (years)	Down-titration	1.0075	1.0032	1.0119	0.0007
Age (years)	Discontinuation	1.0042	1.0003	1.0082	0.0323
Gender at baseline: Male	Down-titration	1.1405	1.0494	1.2396	0.002
Gender at baseline: Male	Discontinuation	1.0591	0.9838	1.1401	0.1268
Time from baseline (years)	Down-titration	0.9989	0.9824	1.0157	0.8956
Time from baseline (years)	Discontinuation	1.0048	0.9898	1.0201	0.5324
Time-updated eGFR: < 15 mL/min/1.73m^2	Down-titration	0.4903	0.2546	0.9441	0.033
Time-updated eGFR: < 15 mL/min/1.73m^2	Discontinuation	1.5142	0.9844	2.3292	0.059
Time-updated eGFR: 30 to <45 mL/min/1.73m^2	Down-titration	0.4866	0.4067	0.5821	<.0001
Time-updated eGFR: 30 to <45 mL/min/1.73m^2	Discontinuation	0.5103	0.4372	0.5957	<.0001
Time-updated eGFR: 45 to <60 mL/min/1.73m^2	Down-titration	0.4981	0.4284	0.5791	<.0001
Time-updated eGFR: 45 to <60 mL/min/1.73m^2	Discontinuation	0.4956	0.4346	0.5652	<.0001
Time-updated eGFR: ≥ 60 mL/min/1.73m^2	Down-titration	0.7037	0.6013	0.8235	<.0001
Time-updated eGFR: ≥ 60 mL/min/1.73m^2	Discontinuation	0.6534	0.5686	0.7510	<.0001
Time-updated blood pressure: Low	Down-titration	1.9071	1.6995	2.1401	<.0001
Time-updated blood pressure: Low	Discontinuation	1.2453	1.1143	1.3917	0.0001
Time-updated blood pressure: Normal	Down-titration	1.3213	1.2062	1.4474	<.0001
Time-updated blood pressure: Normal	Discontinuation	1.3408	1.2400	1.4499	<.0001
Time-updated blood pressure: Pre-High	Down-titration	5.7110	3.8741	8.4189	<.0001
Time-updated blood pressure: Pre-High	Discontinuation	1.9494	1.1681	3.2532	0.0107
Concomitant diuretics: Yes	Down-titration	0.8136	0.1133	5.8443	0.8375
Concomitant diuretics: Yes	Discontinuation	0.5725	0.0766	4.2787	0.5868
Concomitant NSAIDS: Yes	Down-titration	0.0006	0.0004	0.0012	<.0001
Concomitant NSAIDS: Yes	Discontinuation	2.1920	0.2835	16.9465	0.452
Concomitant Betablockers: Yes	Down-titration	2.0168	0.6213	6.5461	0.2428
Concomitant Betablockers: Yes	Discontinuation	0.9622	0.2256	4.1037	0.9585
Concomitant RAASi: Yes	Down-titration	1.4384	1.2698	1.6293	<.0001
Concomitant RAASi: Yes	Discontinuation	3.0177	2.7104	3.3599	<.0001

CI: confidence interval; eGFR: estimated glomerular filtration rate; HF: heart failure; MACE: major adverse cardiac event; OR: odds ratio; RAASi: renin-angiotensin-aldosterone system inhibitor; SE: standard error.
A one-unit change in explanatory variables increase the log odds of down-titration or discontinuation by the value of the corresponding estimate.

Table S3. Model output for dose modification of RAASi, stratified by serum K+ threshold for HF cohort.

Explanatory variable	Outcome	OR	95% lower CI	95% upper CI	P-value
**Incidence of down-titration or discontinuation: Hyperkalaemia threshold: 5.0 mmol/L**					
Serum potassium: ≥5.0 mmol/L	Down-titration	1.3250	1.0832	1.6207	0.0062
Serum potassium: ≥5.0 mmol/L	Discontinuation	1.1341	0.9163	1.4036	0.2479
Drug type: ARB	Down-titration	0.3855	0.3053	0.4869	<.0001
Drug type: ARB	Discontinuation	1.1037	0.9043	1.3472	0.3322
Drug type: MRA	Down-titration	1.1521	0.9369	1.4168	0.1795
Drug type: MRA	Discontinuation	0.7204	0.5507	0.9423	0.0167
Age (years)	Down-titration	1.0088	1.0012	1.0166	0.022
Age (years)	Discontinuation	1.0126	1.0045	1.0207	0.0025
Gender at baseline: Male	Down-titration	1.4118	1.1667	1.7085	0.0004
Gender at baseline: Male	Discontinuation	1.0280	0.8473	1.2471	0.7794
Time from baseline (years)	Down-titration	0.8974	0.8501	0.9472	<.0001
Time from baseline (years)	Discontinuation	0.9251	0.8754	0.9777	0.0059
Time-updated eGFR: < 15 mL/min/1.73m^2	Down-titration	3.1522	1.0667	9.3146	0.0378
Time-updated eGFR: < 15 mL/min/1.73m^2	Discontinuation	5.0576	1.7513	14.6063	0.0027
Time-updated eGFR: 30 to <45 mL/min/1.73m^2	Down-titration	0.8873	0.5015	1.5698	0.6811
Time-updated eGFR: 30 to <45 mL/min/1.73m^2	Discontinuation	0.5572	0.3565	0.8708	0.0103
Time-updated eGFR: 45 to <60 mL/min/1.73m^2	Down-titration	1.0342	0.5775	1.8521	0.9101
Time-updated eGFR: 45 to <60 mL/min/1.73m^2	Discontinuation	0.5726	0.3604	0.9098	0.0183
Time-updated eGFR: ≥ 60 mL/min/1.73m^2	Down-titration	1.0901	0.6087	1.9523	0.7716
Time-updated eGFR: ≥ 60 mL/min/1.73m^2	Discontinuation	0.5606	0.3377	0.9304	0.0252
Time-updated blood pressure: Low	Down-titration	1.5510	1.2625	1.9054	<.0001
Time-updated blood pressure: Low	Discontinuation	1.0048	0.8123	1.2429	0.9649
Time-updated blood pressure: Normal	Down-titration	1.0369	0.8278	1.2988	0.7529
Time-updated blood pressure: Normal	Discontinuation	1.0169	0.8216	1.2587	0.8773
Time-updated blood pressure: Pre-High	Down-titration	3.3364	1.9145	5.8146	<.0001
Time-updated blood pressure: Pre-High	Discontinuation	1.5633	0.7125	3.4300	0.2651
Concomitant diuretics: Yes	Down-titration	1.2880	0.9917	1.6729	0.0579
Concomitant diuretics: Yes	Discontinuation	0.7465	0.5715	0.9751	0.0319
Concomitant NSAIDS: Yes	Down-titration	1.0712	0.7411	1.5485	0.7145
Concomitant NSAIDS: Yes	Discontinuation	1.0520	0.7122	1.5539	0.7989
Concomitant Betablockers: Yes	Down-titration	1.0592	0.8817	1.2725	0.5391
Concomitant Betablockers: Yes	Discontinuation	0.8557	0.7117	1.0288	0.0975
Concomitant RAASi: Yes	Down-titration	1.1522	0.9464	1.4028	0.1582
Concomitant RAASi: Yes	Discontinuation	1.4110	1.1405	1.7457	0.0015
**Incidence of down-titration or discontinuation: Hyperkalaemia threshold: 5.5 mmol/L**					
Serum potassium: ≥5.5 mmol/L	Down-titration	1.9278	1.4125	2.6312	<.0001
Serum potassium: ≥5.5 mmol/L	Discontinuation	1.6960	1.2233	2.3514	0.0015
Drug type: ARB	Down-titration	0.3852	0.3049	0.4866	<.0001
Drug type: ARB	Discontinuation	1.1011	0.9021	1.3440	0.3435
Drug type: MRA	Down-titration	1.1563	0.9399	1.4224	0.1697
Drug type: MRA	Discontinuation	0.7223	0.5521	0.9450	0.0176
Age (years)	Down-titration	1.0089	1.0013	1.0167	0.0217
Age (years)	Discontinuation	1.0124	1.0041	1.0207	0.003
Gender at baseline: Male	Down-titration	1.4130	1.1676	1.7099	0.0004
Gender at baseline: Male	Discontinuation	1.0273	0.8469	1.2460	0.785
Time from baseline (years)	Down-titration	0.8980	0.8509	0.9477	<.0001
Time from baseline (years)	Discontinuation	0.9249	0.8750	0.9776	0.0057
Time-updated eGFR: < 15 mL/min/1.73m^2	Down-titration	3.0508	1.0614	8.7693	0.0384
Time-updated eGFR: < 15 mL/min/1.73m^2	Discontinuation	4.8235	1.6507	14.0949	0.004
Time-updated eGFR: 30 to <45 mL/min/1.73m^2	Down-titration	0.8962	0.5024	1.5987	0.7105
Time-updated eGFR: 30 to <45 mL/min/1.73m^2	Discontinuation	0.5737	0.3679	0.8946	0.0142
Time-updated eGFR: 45 to <60 mL/min/1.73m^2	Down-titration	1.0519	0.5829	1.8983	0.8664
Time-updated eGFR: 45 to <60 mL/min/1.73m^2	Discontinuation	0.5899	0.3718	0.9359	0.025
Time-updated eGFR: ≥ 60 mL/min/1.73m^2	Down-titration	1.0873	0.6025	1.9622	0.7811

Time-updated eGFR: ≥ 60 mL/min/1.73m^2	Discontinuation	0.5623	0.3386	0.9336	0.026
Time-updated blood pressure: Low	Down-titration	1.5583	1.2682	1.9148	<.0001
Time-updated blood pressure: Low	Discontinuation	1.0144	0.8196	1.2555	0.8954
Time-updated blood pressure: Normal	Down-titration	1.0371	0.8278	1.2993	0.7518
Time-updated blood pressure: Normal	Discontinuation	1.0209	0.8247	1.2638	0.8494
Time-updated blood pressure: Pre-High	Down-titration	3.3923	1.9480	5.9072	<.0001
Time-updated blood pressure: Pre-High	Discontinuation	1.5987	0.7304	3.4995	0.2404
Concomitant diuretics: Yes	Down-titration	1.2783	0.9845	1.6596	0.0653
Concomitant diuretics: Yes	Discontinuation	0.7436	0.5691	0.9715	0.0298
Concomitant NSAIDS: Yes	Down-titration	1.0726	0.7438	1.5469	0.7076
Concomitant NSAIDS: Yes	Discontinuation	1.0549	0.7149	1.5565	0.788
Concomitant Betablockers: Yes	Down-titration	1.0648	0.8863	1.2792	0.5023
Concomitant Betablockers: Yes	Discontinuation	0.8575	0.7134	1.0308	0.1017
Concomitant RAASi: Yes	Down-titration	1.1530	0.9476	1.4030	0.1549
Concomitant RAASi: Yes	Discontinuation	1.3972	1.1280	1.7307	0.0022
**Incidence of down-titration or discontinuation: Hyperkalaemia threshold: 6.0 mmol/L**					
Serum potassium: ≥6.0 mmol/L	Down-titration	3.1912	1.8593	5.4771	<.0001
Serum potassium: ≥6.0 mmol/L	Discontinuation	2.7357	1.5312	4.8879	0.0007
Drug type: ARB	Down-titration	0.3867	0.3060	0.4887	<.0001
Drug type: ARB	Discontinuation	1.1063	0.9062	1.3506	0.3213
Drug type: MRA	Down-titration	1.1589	0.9423	1.4254	0.1624
Drug type: MRA	Discontinuation	0.7240	0.5535	0.9471	0.0185
Age (years)	Down-titration	1.0090	1.0016	1.0166	0.0186
Age (years)	Discontinuation	1.0125	1.0044	1.0206	0.0026
Gender at baseline: Male	Down-titration	1.4152	1.1700	1.7119	0.0003
Gender at baseline: Male	Discontinuation	1.0263	0.8461	1.2449	0.7915
Time from baseline (years)	Down-titration	0.9001	0.8531	0.9498	0.0001
Time from baseline (years)	Discontinuation	0.9270	0.8772	0.9797	0.0072
Time-updated eGFR: < 15 mL/min/1.73m^2	Down-titration	3.2333	1.1458	9.1242	0.0266
Time-updated eGFR: < 15 mL/min/1.73m^2	Discontinuation	5.2200	1.8454	14.7653	0.0018
Time-updated eGFR: 30 to <45 mL/min/1.73m^2	Down-titration	0.8770	0.4941	1.5569	0.6541
Time-updated eGFR: 30 to <45 mL/min/1.73m^2	Discontinuation	0.5754	0.3677	0.9005	0.0156
Time-updated eGFR: 45 to <60 mL/min/1.73m^2	Down-titration	1.0346	0.5757	1.8594	0.9095
Time-updated eGFR: 45 to <60 mL/min/1.73m^2	Discontinuation	0.5935	0.3733	0.9434	0.0274
Time-updated eGFR: ≥ 60 mL/min/1.73m^2	Down-titration	1.0819	0.6021	1.9440	0.7923
Time-updated eGFR: ≥ 60 mL/min/1.73m^2	Discontinuation	0.5699	0.3431	0.9466	0.0299
Time-updated blood pressure: Low	Down-titration	1.5597	1.2693	1.9165	<.0001
Time-updated blood pressure: Low	Discontinuation	1.0134	0.8191	1.2538	0.9026
Time-updated blood pressure: Normal	Down-titration	1.0394	0.8296	1.3021	0.7373
Time-updated blood pressure: Normal	Discontinuation	1.0248	0.8278	1.2686	0.8223
Time-updated blood pressure: Pre-High	Down-titration	3.4264	1.9584	5.9947	<.0001
Time-updated blood pressure: Pre-High	Discontinuation	1.6048	0.7317	3.5197	0.2378
Concomitant diuretics: Yes	Down-titration	1.2697	0.9782	1.6482	0.0728
Concomitant diuretics: Yes	Discontinuation	0.7413	0.5675	0.9684	0.0282
Concomitant NSAIDS: Yes	Down-titration	1.0753	0.7450	1.5519	0.6979
Concomitant NSAIDS: Yes	Discontinuation	1.0576	0.7167	1.5606	0.7779
Concomitant Betablockers: Yes	Down-titration	1.0685	0.8896	1.2835	0.4787
Concomitant Betablockers: Yes	Discontinuation	0.8605	0.7159	1.0344	0.1097
Concomitant RAASi: Yes	Down-titration	1.1654	0.9578	1.4181	0.126
Concomitant RAASi: Yes	Discontinuation	1.4055	1.1356	1.7396	0.0018

CI: confidence interval; eGFR: estimated glomerular filtration rate; HF: heart failure; MACE: major adverse cardiac event; OR: odds ratio; RAASi: renin-angiotensin-aldosterone system inhibitor; SE: standard error.
A one-unit change in explanatory variables increase the log odds of down-titration or discontinuation by the value of the corresponding estimate.

Table S4. Model output for adverse outcomes stratified by RAASi dose within interval outcome occurred for the CKD cohort.

Explanatory variable	IRR	95% lower CI	95% upper CI	P-value
**Incidence of death**				
Mean RAASi dose in interval: <50%	5.6013	5.2878	5.9333	<0.0001
Time-updated BMI (kg/m2)	0.9847	0.9806	0.9889	<0.0001
Time-updated blood pressure: Low	2.0211	1.6524	2.4722	<0.0001
Time-updated blood pressure: Pre-high	0.6307	0.6013	0.6616	<0.0001
Time-updated blood pressure: High	0.5256	0.4992	0.5533	<0.0001
Time-updated serum potassium (mmol/L)	0.9763	0.9379	1.0163	0.2418
Time-updated phosphorous (mmol/L)	1.0039	0.9964	1.0114	0.3115
Time-updated eGFR (mL/min/1.73m2)	0.9849	0.9831	0.9866	<0.0001
Time-updated cholesterol (mmol/L)	0.7701	0.7539	0.7867	<0.0001
Time-updated WBC (x109/L)	1.0949	1.0879	1.1019	<0.0001
Time-updated OADs usage: Yes	0.6545	0.6052	0.7079	<0.0001
Time-updated CCB usage: Yes	0.7337	0.6999	0.7691	<0.0001
Time-updated diuretic usage: Yes	0.8486	0.8131	0.8858	<0.0001
Time-updated beta blockers usage: Yes	0.7006	0.6692	0.7333	<0.0001
Time-updated statins usage: Yes	0.3484	0.3328	0.3647	<0.0001
History of HF prior to interval: Yes	1.8325	1.7172	1.9555	<0.0001
History of MACE prior to interval: Yes	1.7810	1.7026	1.8630	<0.0001
History of PVD prior to interval: Yes	1.5806	1.4627	1.7080	<0.0001
History of dementia prior to interval: Yes	1.7772	1.6545	1.9090	<0.0001
History of diabetes (without chronic) prior to interval: Yes	1.7260	1.6275	1.8304	<0.0001
History of cancer prior to interval: Yes	1.9352	1.8512	2.0230	<0.0001
History of metatumour prior to interval: Yes	1.8557	1.7191	2.0032	<0.0001
Age (years)	1.0588	1.0560	1.0617	<0.0001
Smoker at baseline: Yes	1.4518	1.3722	1.5361	<0.0001
Time since baseline (days)	0.9999	0.9999	0.9999	<0.0001
**Incidence of MACE**				
Mean RAASi dose in interval: <50%	1.6039	1.5528	1.6568	<0.0001
Time-updated BMI (kg/m^2)	0.9984	0.9953	1.0015	0.2986
Time-updated blood pressure: Low	1.2004	1.0083	1.4292	0.0401
Time-updated blood pressure: Pre-high	0.8816	0.8482	0.9162	<0.0001
Time-updated blood pressure: High	0.9547	0.9159	0.9951	0.0285
Time-updated serum potassium (mmol/L)	0.8977	0.8726	0.9235	<0.0001
Time updated cholesterol (mmol/L)	0.9924	0.9771	1.0079	0.3327
Time-updated WBC (x10^9/L)	1.0307	1.0246	1.0370	<0.0001
Time-updated diuretic use: Yes	1.1718	1.1309	1.2141	<0.0001
Time-updated beta blockers use: Yes	2.0226	1.9486	2.0994	<0.0001
Time-updated statins usage: Yes	1.2579	1.2101	1.3076	<0.0001
Time-updated bronchodilators usage: Yes	1.2741	1.2212	1.3293	<0.0001
History of HF prior to interval: Yes	12.4495	11.6996	13.2475	<0.0001
History of MACE prior to interval: Yes (one quarter lagged)	0.5394	0.5068	0.5742	<0.0001
Age (years)	1.0328	1.0307	1.0349	<0.0001
Gender at baseline (Male)	1.1619	1.1215	1.2038	<0.0001
Time since baseline (days)	0.9998	0.9998	0.9998	<0.0001

BMI: body mass index; CI: confidence interval; eGFR: estimated glomerular filtration rate; HF: heart failure; IRR: incident rate ratios; MACE: major adverse cardiac event; RAASi: renin-angiotensin-aldosterone system inhibitor; SE: standard error.

A one-unit change in explanatory variables increased the expected incidence of death or MACE changes by the value of the corresponding estimate.

Table S5. Model output for adverse outcomes stratified by RAASi dose within interval outcome occurred for the HF cohort.

Explanatory variable	IRR	95% lower CI	95% upper CI	P-value
**Incidence of death**				
Mean RAASi dose in interval: <50%	7.3356	6.3463	8.4792	<0.0001
Time-updated BMI (kg/m^2)	0.9807	0.9732	0.9883	<0.0001
Time-updated blood pressure: Low	2.0305	1.6685	2.4711	<0.0001
Time-updated blood pressure: Pre-high	0.6533	0.6007	0.7106	<0.0001
Time-updated blood pressure: High	0.5727	0.5180	0.6332	<0.0001
Time-updated phosphorous (mmol/L)	1.2850	1.1512	1.4344	<0.0001
Time-updated serum potassium(mmol/L)	0.8546	0.7918	0.9224	0.0001
Time-updated eGFR (mL/min/1.73m^2)	0.9894	0.9870	0.9918	<0.0001
Time-updated cholesterol (mmol/L)	0.8017	0.7695	0.8352	<0.0001
Time-updated WBC (x10^9/L)	1.0793	1.0641	1.0947	<0.0001
Time-updated CCB use: Yes	0.6687	0.5778	0.7740	<0.0001
Time-updated diuretic use: Yes	0.6687	0.6132	0.7292	<0.0001
Time-updated beta blockers use: Yes	0.5238	0.4813	0.5699	<0.0001
Time-updated statins use: Yes	0.4077	0.3726	0.4460	<0.0001
History of MACE prior to interval: Yes	1.2100	1.1129	1.3156	<0.0001
History of PVD prior to interval: Yes	1.4277	1.2285	1.6591	<0.0001
History of dementia prior to interval: Yes	1.3870	1.1860	1.6220	<0.0001
History of diabetes (without chronic) prior to interval: Yes	1.3673	1.2346	1.5143	<0.0001
History of cancer prior to interval: Yes	1.4619	1.3310	1.6057	<0.0001
History of metatumour prior to interval: Yes	1.6344	1.3650	1.9570	<0.0001
Age (years)	1.0376	1.0328	1.0424	<0.0001
Gender at baseline: Male	1.1232	1.0318	1.2226	0.0073
Smoker at baseline: Yes	1.1287	1.0094	1.2621	0.0337
Time since baseline (days)	0.9998	0.9998	0.9999	<0.0001
**Incidence of MACE**				
Mean RAASi dose in interval: <50%	1.8471	1.7132	1.9914	<0.0001
Time-updated BMI (kg/m2)	0.9920	0.9862	0.9977	0.0061
Time-updated serum potassium (mmol/L)	0.8077	0.7560	0.8629	<0.0001
Time updated eGFR (mL/min/1.73m2)	0.9953	0.9931	0.9975	<0.0001
Age (years)	1.0070	1.0036	1.0103	<0.0001
Time since baseline (days)	0.9994	0.9993	0.9995	<0.0001

BMI: body mass index; CCB: calcium channel blockers; CI: confidence interval; eGFR: estimated glomerular filtration rate; HF: heart failure; IRR: incident rate ratios; MACE: major adverse cardiac event; RAASi: renin-angiotensin-aldosterone system inhibitor; SE: standard error; WBC: white blood cell.

A one-unit change in explanatory variables increased the expected incidence of death or MACE changes by the value of the corresponding estimate.

Table S6. Model output for survival analysis of adverse outcomes stratified by majority RAASi dose over the follow up for CKD cohort.

Explanatory variable	HR	95% lower CI	95% upper CI	P-value
**Death**				
Mean RAASi dose in interval: <50%	1.2074	1.1737	1.2421	<0.0001
Mean RAASi dose in interval: ≥50%	0.6829	0.6599	0.7068	<0.0001
Baseline age (years)	1.0942	1.0926	1.0957	<0.0001
Gender at baseline: Male	1.3616	1.3292	1.3947	<0.0001
Baseline smoker: Yes	1.7042	1.6475	1.7629	<0.0001
Baseline BMI (kg/m^2)	0.9827	0.9803	0.9852	<0.0001
History of diabetes	1.4735	1.4248	1.5238	<0.0001
**MACE**				
Mean RAASi dose in interval: <50%	2.2846	2.2175	2.3538	<0.0001
Mean RAASi dose in interval: ≥50%	1.6882	1.6330	1.7453	<0.0001
Baseline age (years)	1.0551	1.0536	1.0565	<0.0001
Gender at baseline: Male	1.3624	1.3282	1.3976	<0.0001
Baseline smoker: Yes	1.1438	1.1002	1.1891	<0.0001
Baseline BMI (kg/m^2)	1.0045	1.0020	1.0070	0.0004
History of diabetes	1.1251	1.0859	1.1657	<0.0001

BMI: body mass index; CI: confidence interval; HR: hazard ratio; MACE: major adverse cardiac event; RAASi: renin-angiotensin-aldosterone system inhibitor; SE: standard error.
A one-unit change in explanatory variables increased the risk of death or MACE by the value of the corresponding hazard ratio.

Table S7. Model output for survival analysis of adverse outcomes stratified by majority achieved RAASi dose over the follow up for HF cohort.

Explanatory variable	HR	95% lower CI	95% upper CI	P-value
**Death**				
Mean RAASi dose in interval: <50%	1.0856	1.0180	1.1577	0.0122
Mean RAASi dose in interval: ≥50%	0.5021	0.4568	0.5517	<0.0001
Baseline age (years)	1.0618	1.0584	1.0652	<0.0001
Gender at baseline: Male	1.1285	1.0618	1.1994	0.0001
Baseline smoker: Yes	1.6205	1.4903	1.7622	<0.0001
Baseline BMI (kg/m^2)	0.9908	0.9855	0.9962	0.0008
History of diabetes	1.1875	1.0901	1.2935	0.0001
**MACE**				
Mean RAASi dose in interval: <50%	3.7225	3.4179	4.0542	<0.0001
Mean RAASi dose in interval: ≥50%	3.1694	2.8766	3.4920	<0.0001
Baseline age (years)	1.0175	1.0147	1.0202	<0.0001
Gender at baseline: Male	1.1127	1.0448	1.1850	0.0009
Baseline smoker: Yes	1.0787	0.9930	1.1718	0.0730
Baseline BMI (kg/m^2)	1.0066	1.0016	1.0117	0.0102
History of diabetes	1.0345	0.9510	1.1253	0.4296

BMI: body mass index; CI: confidence interval; HR: hazard ratio; MACE: major adverse cardiac event; RAASi: renin-angiotensin-aldosterone system inhibitor; SE: standard error.
A one-unit change in explanatory variables increased the risk of death or MACE by the value of the corresponding hazard ratio.

Table S8. Summary of sample size and patient attrition.

Population	CKD cohort		HF cohort	
	N	%	N	%
Patients meeting CKD/HF-specific inclusion criteria identified in the CPRD	191,964	100.00%	21,334	100.00%
Patients not in receipt of any RAASi therapy at any point over the follow-up period: **Final analysed Non-RAASi cohort**	**71,008**	**36.99%**	**6,063**	**28.42%**
Prescriptions for RAASi agents not recommended by ESC 2016 guidelines for treatment of HF	-14,132	-7.36%	-996	-4.67%
RAASi prescriptions with missing/unusable dose information	-6,252	-3.26%	-1,162	-5.45%
**Final analysed RAASi cohort**	**100,572**	**52.39%**	**13,113**	**61.47%**

CPRD: Clinical Practice Research Datalink; CKD: chronic kidney disease; ESC: European Society of Cardiology; HF: heart failure; RAASi: Renin-Angiotensin-Aldosterone System Inhibitors

Table S9. Baseline patient demographics, clinical characteristics and clinical histories of CKD and HF patients included in the analysis (RAASi) and patients excluded due to receiving RAASi agents for which a recommended dose was not specified by the European Society of Cardiology guidelines[1], or due to missing/unusable RAASi dose information.

Variable	CKD cohort			HF cohort		
	RAASi (n=100,572)	Excluded patients† (n=20,384)	p-value (ANOVA /χ^2)	RAASi (n=13,113)	Excluded patients† (n=2,157)	p-value (ANOVA /χ^2)
**Baseline* patient demographics and clinical characteristics, mean (SD)**						
Age (years)	73.59 (10.93)	73.21 (11.26)	<0.01‡	72.88 (13.13)	73.55 (13.18)	0.03‡
Female, n (%)	58,277 (57.95%)	11,758 (57.69%)	0.49	5,302 (40.43%)	933 (43.25%)	0.01‡
Current Smoker, n (%)	13,742 (13.66%)	2,739 (13.44%)	0.40	2,469 (18.83%)	391 (18.13%)	0.46
BMI (kg/m^2)	28.91 (5.89)	29.00 (5.89)	0.28	28.47 (6.73)	28.27 (6.65)	0.53
SBP (mmHg)	141.97 (20.79)	140.68 (19.87)	<0.01‡	129.34 (21.78)	129.49 (22.12)	0.82
eGFR (mL/min/1.73m2)	50.85 (10.35)	4.48 (0.53)	<0.01‡	65.14 (17.35)	4.41 (0.55)	<0.01‡
Serum potassium (mEq/L)	4.51 (0.53)	50.70 (8.62)	<0.01‡	4.43 (0.54)	69.2 (15.01)	<0.01‡
Serum phosphorus (mEq/L)	1.14 (1.03)	1.13 (0.22)	0.67	1.15 (0.22)	1.15 (0.22)	0.99
**Clinical history within 5 years prior to initial CKD/HF diagnosis, n (%)**						
Hx diabetes	18,898 (18.79%)	3,957 (19.41%)	0.04‡	2,122 (16.18%)	347 (16.09%)	0.94
Hx MI	4,571 (4.55%)	834 (4.09%)	<0.01‡	1,543 (11.77%)	226 (10.48%)	0.09
Hx PVD	2,994 (2.98%)	567 (2.78%)	0.14	453 (3.45%)	62 (2.87%)	0.19
Hx stroke	6,410 (6.37%)	1,842 (9.04%)	<0.01‡	871 (6.64%)	195 (9.04%)	<0.01‡
Hx arrhythmia	8,769 (8.72%)	1,751 (8.59%)	0.56	3,192 (24.34%)	517 (23.97%)	0.73
Hx CPD	10,240 (10.18%)	1,962 (9.63%)	0.02*	2,020 (15.40%)	319 (14.79%)	0.48
Hx metastatic tumour	2,209 (2.20%)	447 (2.19%)	1.00	231 (1.76%)	35 (1.62%)	0.71
Hx rheumatic disease	3,381 (3.36%)	689 (3.38%)	0.91	374 (2.85%)	60 (2.78%)	0.91
Hx peptic ulcer	899 (0.89%)	182 (0.89%)	<0.01‡	146 (1.11%)	24 (1.11%)	1.00
Hx cancer	8,721 (8.67%)	1,863 (9.14%)	0.03‡	1,349 (10.29%)	230 (10.66%)	0.62
**Baseline* medication usage, n (%)**						
Beta blockers	32,044 (31.86%)	5,979 (29.33%)	<0.01‡	7,957 (60.68%)	1,113 (51.60%)	<0.01‡
Statins	56,138 (55.82%)	10,774 (52.86%)	<0.01‡	7,200 (54.91%)	1,027 (47.61%)	<0.01‡
Bronchodilators	11,566 (11.50%)	2,352 (11.54%)	0.89	2,691 (20.52%)	431 (19.98%)	0.58
Diuretics	49,721 (49.44%)	10,084 (49.47%)	0.94	10,449 (79.68%)	1,406 (65.18%)	<0.01‡
NSAIDs	9,439 (9.39%)	2,675 (13.12%)	<0.01‡	643 (4.90%)	167 (7.74%)	<0.01‡
Calcium channel blockers	33,644 (33.45%)	6,938 (34.04%)	0.11	2,082 (15.88%)	459 (21.28%)	<0.01‡
OADs	13,316 (13.24%)	2,554 (12.53%)	<0.01‡	1,515 (11.55%)	217 (10.06%)	0.05‡

Insulin	3,849 (3.83%)	757 (3.71%)	0.45	445 (3.39%)	65 (3.01%)	0.40

BMI: body mass index; CKD: chronic kidney disease; CPD: cardiopulmonary disease; eGFR: estimated glomerular filtration rate; ESC: European Society of Cardiology; HF: heart failure; Hx: History of; MI: myocardial infarction; NSAIDs: non-steroidal anti-inflammatory drugs; OADs: oral antidiabetics; RAASi: renin-angiotensin-aldosterone system inhibitors; SBP: systolic blood pressure; SD: standard deviation. ANOVA and Chi-squared test were used to evaluate differences between HbA1c groups for continuous and categorical variables, respectively. *Baseline for RAASi patients is time of each patient's first RAASi prescription after their first CKD/HF event, for the excluded patients its time of first CKD/HF event. †Patients excluded due to to being in receipt of RAASi agents for which a recommended dose was not specified by European Society of Cardiology guidelines or due to missing/unusable RAASi dose information. ‡ Indicates significance at $p < 0.05$

Figure S1. Illustrative example of data structuring for estimating associations between renin-angiotensin-aldosterone system inhibitor dose and adverse clinical outcomes.

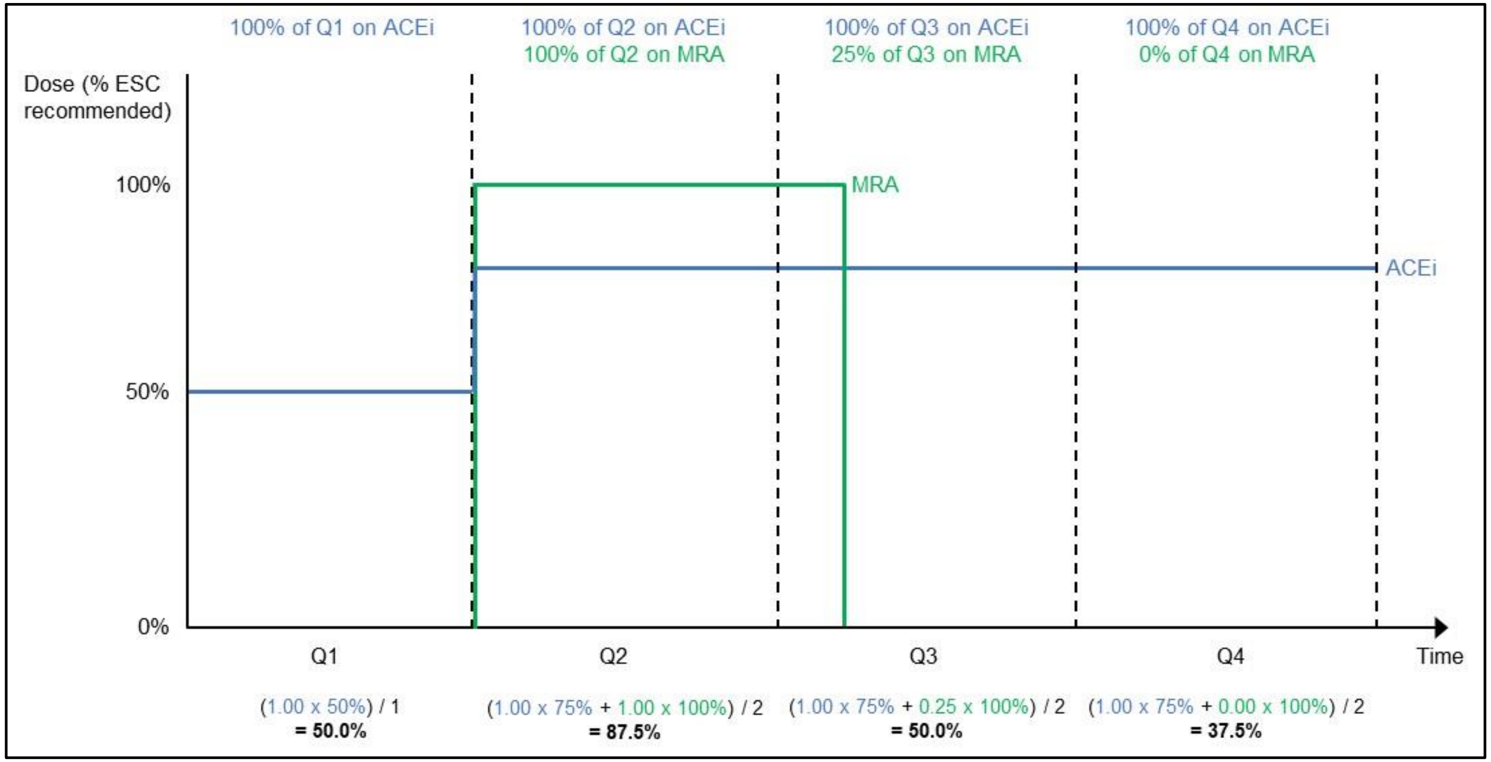

Figure S2. Three stages of the multiple imputation process.

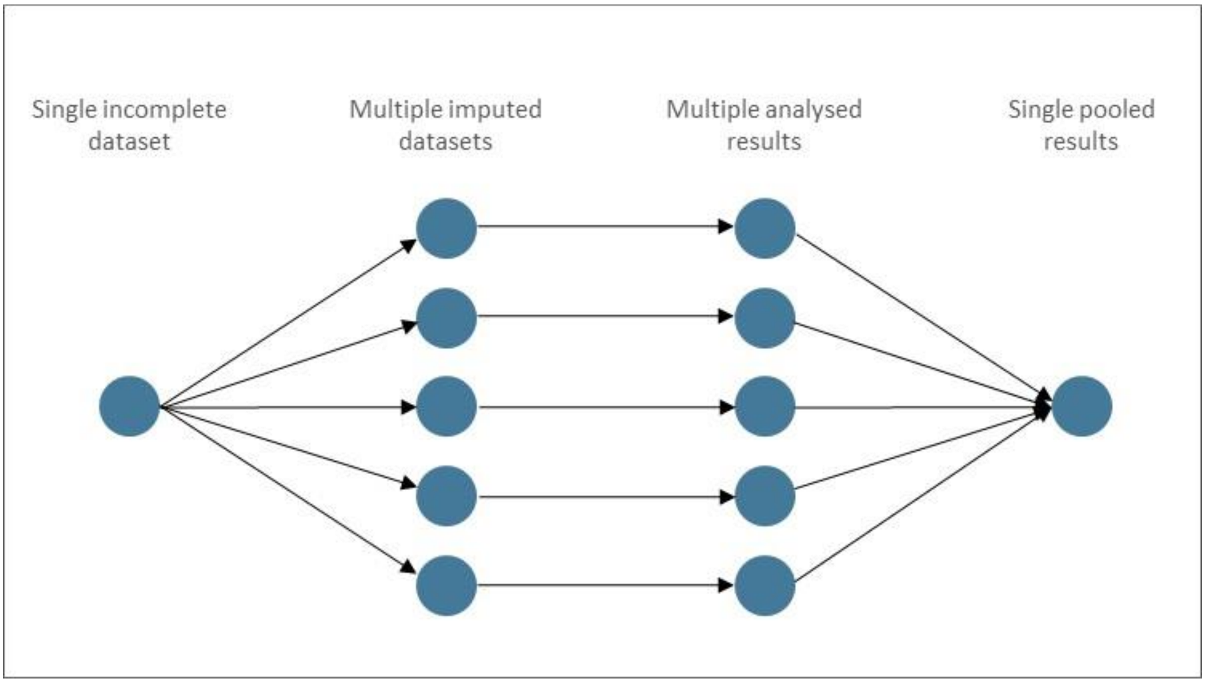

Figure S3. Simple description of the Chained Equations algorithm.

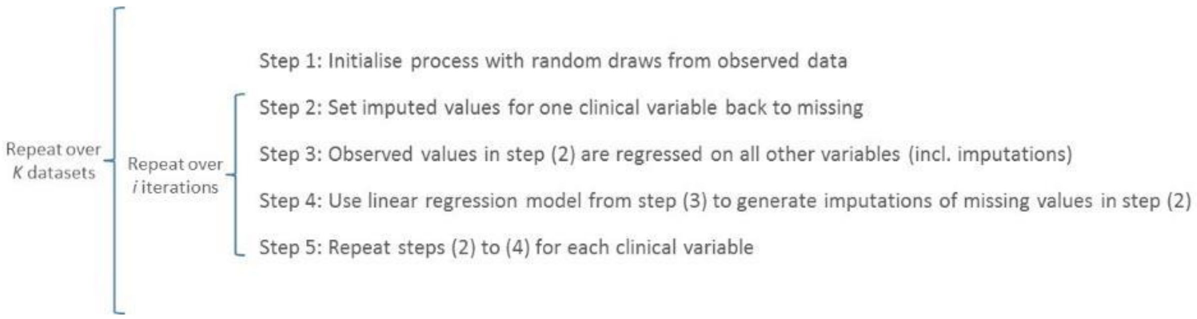

Supplemental References:

1. Ponikowski P, Voors AA, Anker SD, Bueno H, Cleland JGF, Coats AJS, Falk V, Gonzalez-Juanatey JR, Harjola VP, Jankowska EA, Jessup M, Linde C, Nihoyannopoulos P, Parissis JT, Pieske B, Riley JP, Rosano GMC, Ruilope LM, Ruschitzka F, Rutten FH, van der Meer P, ESC Scientific Document Group. 2016 ESC guidelines for the diagnosis and treatment of acute and chronic heart failure: The task force for the diagnosis and treatment of acute and chronic heart failure of the European Society of Cardiology (ESC) developed with the special contribution of the Heart Failure Association (HFA) of the ESC. *Eur Heart J*. 2016;37:2129-2200.
2. Carpenter J, R., Kenward MG. *Missing data in randomised controlled trials: A practical guide*. Birmingham: Health Technology Assessment Methodology Programme; 2007.
3. Marston L, Carpenter JR, Walters KR, Morris RW, Nazareth I, Petersen I. Issues in multiple imputation of missing data for large general practice clinical databases. *Pharmacoepidemiol Drug Saf.* 2010;19:618-626.
4. Sterne JA, White IR, Carlin JB, Spratt M, Royston P, Kenward MG, Wood AM, Carpenter JR. Multiple imputation for missing data in epidemiological and clinical research: Potential and pitfalls. *BMJ (Clinical research ed.)*. 2009;338:b2393.
5. Azur MJ, Stuart EA, Frangakis C, Leaf PJ. Multiple imputation by chained equations: What is it and how does it work? *Int J Methods Psychiatr Res*. 2011;20:40-4.9
6. van Buuren S, Groothuis-Oudshoorn K. Mice: Multivariate imputation by chained equations in R. *J Stat Softw*. 2011;1.
